# Engineering bacterial morphology through MreB remodeling enhances one-carbon metabolism for sustainable bioproduction

**DOI:** 10.1126/sciadv.aej6280

**Published:** 2026-10-28

**Authors:** Yun Chen, Jiahui Peng, Liang Guo, Zaigao Tan

**Affiliations:** ^1^ State Key Laboratory of Microbial Metabolism, Shanghai Jiao Tong University, Shanghai 200240, China.; ^2^ Department of Bioengineering, School of Life Sciences and Biotechnology, Shanghai Jiao Tong University, Shanghai 200240, China.; ^3^ School of Life Science, Shanxi Normal University, Taiyuan, Shanxi 030031, China.

## Abstract

The actin-like cytoskeletal protein MreB controls bacterial cell morphology, yet whether engineered modulation of MreB-associated architecture can improve microbial metabolism remains largely unexplored. Here, we established a chemical-genetic platform in *Escherichia coli* combining an engineered MreB variant (A53R) that remodels cellular morphology with A22-mediated perturbation of MreB dynamics. MreB(A53R) reshaped cell morphology and engaged an RpoE-Cra-GadE regulatory network associated with cellular adaptation, which remodeled electron transport, redirected carbon flux toward NADPH-generating pathways, and reduced intracellular reactive oxygen species (ROS). Consequently, engineered strains exhibited enhanced formaldehyde tolerance and efficient assimilation of methanol and other C1 substrates. Acute A22 treatment recapitulated the morphological alteration but failed to induce metabolic rewiring, revealing a two-stage adaptive response involving immediate physical adaptation associated with cell envelope reinforcement and long-term transcription-dependent metabolic adaptation to persistent morphological remodeling. The analogous V45R mutation in the native methylotroph *Bacillus methanolicus* partially recapitulated the improved C1 utilization phenotype, supporting the transferability of MreB-based morphological engineering. Collectively, these findings establish MreB-mediated morphological engineering as a strategy to couple cellular architecture with central carbon and redox metabolism and provide a framework for engineering robust microbial chassis for C1 bioconversion.

## INTRODUCTION

Bacterial cell morphology is primarily determined by the peptidoglycan cell wall, and the conserved cytoskeletal protein MreB—a prokaryotic homolog of actin—orchestrates cell wall synthesis to maintain the canonical rod shape of most Gram-negative bacteria (*1*). Decades of research have defined MreB as a structural determinant of cell shape, cell division, and membrane organization (*2*). Although emerging evidence suggests that cell geometry influences bacterial physiology, including motility, adhesion, and stress adaptation, whether engineered modulation of MreB-associated cellular architecture can be exploited to improve central metabolism, redox homeostasis, and stress tolerance remains largely unexplored (*3*). Understanding how cytoskeletal regulation influences cellular physiology may reveal alternative principles for rational microbial engineering and cellular reprogramming (*3*).

Microbial conversion of one-carbon (C1) compounds such as methanol, formate, and formaldehyde is a promising route for sustainable biomanufacturing (*4*). However, widespread application of C1 bioconversion is hindered by two major bottlenecks: Formaldehyde, a universal intermediate in nearly all C1 metabolic pathways, causes severe cytotoxicity, DNA-protein crosslinks (DPCs), protein aggregation, and excessive reactive oxygen species (ROS) accumulation; meanwhile, native cellular metabolism creates a redox imbalance incompatible with high-flux C1 assimilation (*5*–*8*). Current solutions depend on iterative metabolic engineering (*9*) or adaptive laboratory evolution (*10*), which are time consuming, strain specific, and difficult to generalize. There is an urgent demand for innovative strategies to globally rewire cellular physiology to adapt to the harsh conditions of C1 metabolism.

To investigate whether MreB-mediated morphological remodeling can influence cellular adaptation, we adopted a chemical-genetic strategy combining an engineered MreB variant and small-molecule perturbation. This approach enabled us to distinguish immediate morphological responses from sustained physiological adaptation associated with altered MreB activity. We constructed an engineered MreB variant (A53R) that remodels bacterial morphology and alters MreB-associated cellular physiology, alongside the well-characterized reversible MreB perturbation compound A22 (*11*) which disrupts MreB-associated cytoskeletal dynamics. This dual chemical-genetic system enables us to distinguish morphological responses from longer-term metabolic adaptation and investigate the physiological consequences of altered MreB activity. We found that the MreB A53R mutation transforms *Escherichia coli* from long rods to short rods and is associated with activation of an RpoE-Cra-GadE stress-responsive regulatory module. This regulatory module is associated with remodeling of electron transport processes, redistribution of carbon flux toward reduced form of nicotinamide adenine dinucleotide phosphate (NADP^+^) (NADPH)–generating pathways, and reduced oxidative stress, thereby greatly improving formaldehyde tolerance and enabling efficient utilization of diverse C1 substrates.

Phenotypic differences between genetic modification and acute A22 treatment reveal a two-stage adaptive model: a rapid envelope-associated protective response and a sustained transcription-dependent metabolic adaptation. Notably, a comparable V45R mutation in the native methylotroph *Bacillus methanolicus* partially recapitulated the improved C1 utilization phenotype, supporting the transferability of MreB-based morphological engineering across bacterial hosts. Our work establishes MreB-mediated morphological engineering as an engineerable strategy that links bacterial cell architecture with carbon and redox metabolism. Beyond expanding our understanding of cytoskeleton-associated physiological adaptation, this study provides a morphology-guided strategy for engineering stress-resistant microbial cell factories and improving C1 bioconversion.

## RESULTS

### An engineered MreB(A53R) allele remodels cell morphology and enhances formaldehyde tolerance

To investigate whether engineered modulation of MreB can influence cellular resilience to C1-associated stress, we targeted *mreB* in *E. coli*. Because *mreB* is essential (*12*), we integrated a single chromosomal copy under the control of a constitutive M1-93 promoter (*13*) at the *pta* locus (Fig. 1B) (*14*), generating strain MB1 (MG1655, *pta*::M1-93-*mreB*_WT_). The *pta* gene alone was deleted in the control strain MB0 (MG1655, Δ*pta*). MB1 showed no increase in formaldehyde tolerance (Fig. 1C).

**Fig. 1.**
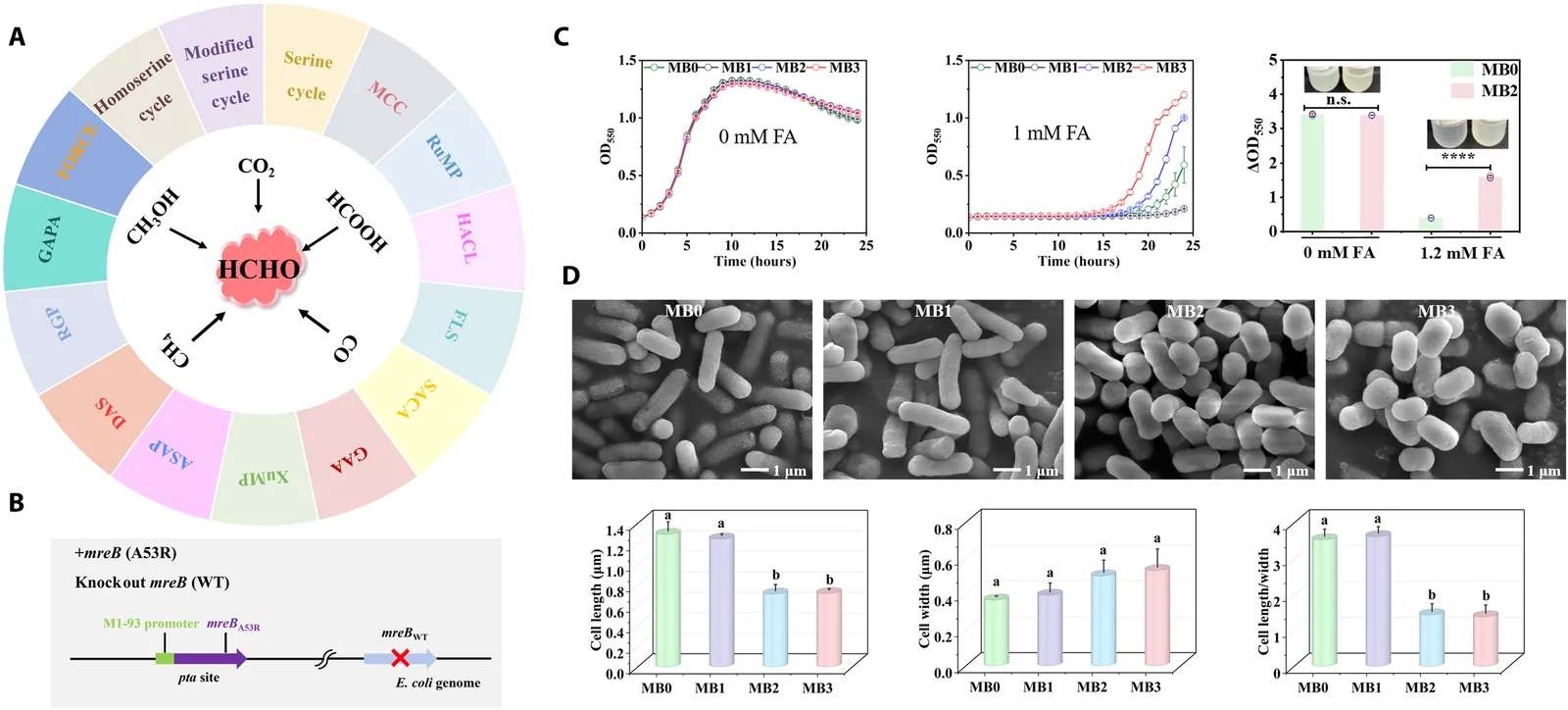
An MreB(A53R) chemical genetic allele alters cell shape and confers formaldehyde tolerance. (**A**) Formaldehyde (HCHO) serves as a key intermediate in methylotrophic metabolism. It plays a central role across various methylotrophic pathways. RuMP, ribulose monophosphate; HACL, 2-hydroxyacyl-CoA-lyase pathway; FLS, formolase pathway; SACA, synthetic acetyl-CoA pathway; GAA, glycolaldehyde assimilation pathway; XuMP, xylulose monophosphate; ASAP, artificial starch anabolic pathway; DAS, dihydroxyacetone synthase pathway; RGP, reductive glycine pathway; GAPA, glycolaldehyde-allose 6-phosphate assimilation pathway; FORCE, formyl-CoA elongation pathway; MCC, methanol condensation cycle. (**B**) Schematic illustrating the engineering strategy. An additional copy of the *mreB* gene, under the control of the strong constitutive promoter M1-93, was integrated into the chromosome at the *pta* locus. Strain MB1 carries the wild-type (WT) *mreB*; strain MB2 harbors the A53R mutation; and strain MB3 has the original WT *mreB* deleted from the MB2 background. (**C**) The A53R mutation confers tolerance to formaldehyde. The left two assays were performed in clear-bottom 96-well plates at 37°C with an initial pH of 7.0, whereas the right assay was performed in a 50-ml tube. “n.s.” means no significance, and “****” means *P* ≤ 0.0001, unpaired two-tailed Student’s *t* test or appropriate post hoc comparison. (**D**) Scanning electron microscopy (SEM) images of strains MB0 to MB3, along with their length, width, and length-to-width ratio. Quantification of cell length, width, and length/width ratio. Different lowercase letters (a, b, and c) indicate significant differences [*P* < 0.05, one-way analysis of variance (ANOVA) with Tukey’s honest significant difference (HSD) post hoc test]; same letter denotes no significant difference.

We next introduced a panel of previously reported *mreB* variants associated with altered cell morphology (*1*). Strains carrying these *mreB* alleles were assayed for growth in Mops medium with 2% glucose and increasing formaldehyde concentrations. Most mutants showed no improvement, but an A53R substitution that had not previously been evaluated in the context of C1-stress adaptation—introduced in strain MB2 (MG1655, *pta*::M1-93-*mreB*_A53R_)—conferred a marked increase in formaldehyde tolerance. In the presence of 1 mM formaldehyde, the specific growth rate of MB2 was 0.31 hour^−1^, a 63% increase over MB0 (0.19 hour^−1^, *P* < 0.01) (Fig. 1C).

We confirmed this phenotype in larger culture volumes. In 50-ml tube cultures with 1.2 mM formaldehyde, MB0 grew poorly, with optical density at 500 nm (OD_500_) rising only from 0.10 to 0.5 over 24 hours (ΔOD_500_ = 0.4). In contrast, MB2 reached an OD_500_ of 1.7 (ΔOD_500_ = 1.6), a fourfold greater increase (Fig. 1C). Deleting the native *mreB* in the MB2 background (strain MB3) (Fig. 1B) further enhanced formaldehyde tolerance (Fig. 1C), suggesting that the engineered A53R allele is sufficient to generate the morphological state associated with improved formaldehyde tolerance.

Scanning electron microscopy (SEM) revealed that MB1 (overexpressing wild-type MreB) retained the typical long-rod morphology of MB0 (Fig. 1D). By contrast, MB2 and MB3 exhibited a notable morphological change: Cells became shorter and wider (Fig. 1D). Quantitative analysis showed that the mean cell length of MB3 was 0.71 μm, only 55% of that of MB0 (1.29 μm), while the mean width increased from 0.37 μm (MB0) to 0.53 μm (MB3), a 43% increase (Fig. 1D). Consequently, the length-to-width ratio of MB3 was 39% of that of MB0 (Fig. 1D). Thus, a single amino acid substitution in MreB is sufficient to remodel *E. coli* morphology from a long-rod to a short-rod state and enhance formaldehyde tolerance.

### The MreB(A53R) allele remodels cell envelope properties and enhances protection against formaldehyde-induced damage

To understand how the MreB A53R-induced morphological change enhances formaldehyde tolerance, we examined the physical properties of the cell envelope (*15*). Atomic force microscopy (AFM) of isolated cell walls showed that the peptidoglycan layer of the MreB(A53R)-engineered strain MB3 exhibited a modest increase in peptidoglycan thickness compared with MB0 (Fig. 2A), consistent with the established association between MreB-dependent cell architecture and peptidoglycan organization (*16*).

**Fig. 2.**
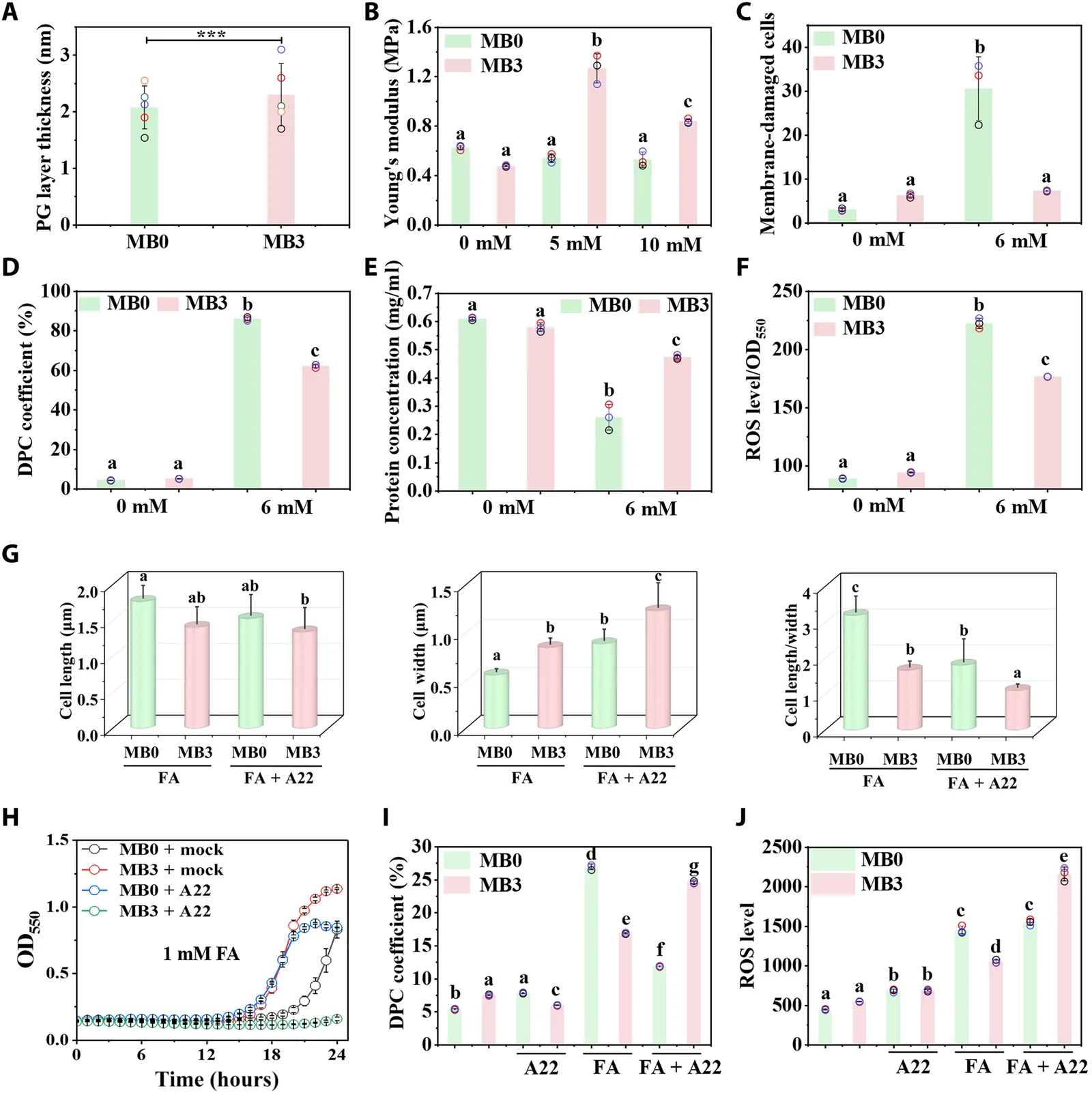
Performance of MreB(A53R) and A22 in formaldehyde tolerance. (**A**) Peptidoglycan (PG) layer thickness measured by AFM. ***P < 0.001 (two-tailed unpaired Student’s *t* test). (**B**) Young’s modulus (cell stiffness) under different formaldehyde concentrations. Different lowercase letters (a, b, and c) indicate significant differences (*P* < 0.05, one-way ANOVA with Tukey’s HSD post hoc test). (**C**) Membrane permeability assessed by SYTOX Green staining. Different letters (a and b) denote *P* < 0.05 (ANOVA + Tukey’s HSD). (**D**) DPC coefficient. Different letters (a, b, c, and d) indicate significant differences (*P* < 0.05, ANOVA + Tukey’s HSD). (**E**) Intracellular soluble protein levels. Different letters (a, b, and c) indicate *P* < 0.05 (ANOVA + Tukey’s HSD). (**F**) Intracellular ROS levels (DCFH-DA assay). Different letters (a, b, and c) indicate *P* < 0.05 (ANOVA + Tukey’s HSD). (**G**) Cell dimensions of MB0 and MB3 with or without A22 (8 mg/liter). Different letters (a, b, and c) indicate *P* < 0.05 (ANOVA + Tukey’s HSD). (**H**) Growth curves in 1 mM formaldehyde (Mops + 2% glucose, 96-well plate). Data are mean ± SD (*n* = 3). (**I**) DPC coefficient with/without A22. Different letters (a to g) denote significant differences (*P* < 0.05, ANOVA + Tukey’s HSD). (**J**) ROS levels with/without A22. Different letters (a to e) denote *P* < 0.05 (ANOVA + Tukey’s HSD). All panels: *n* = 3 biologically independent samples. Error bars represent SD.

We next measured cell stiffness using AFM (*17*). In the absence of formaldehyde, MB0 and MB3 had similar Young’s moduli (*18*). Upon exposure to 5 mM formaldehyde, however, the surface of MB0 developed wrinkles and indentations, whereas MB3 remained smooth (Fig. 2B). The Young’s modulus of MB3 was 2.4-fold higher than that of MB0 under this condition (Fig. 2B). At 10 mM formaldehyde, MB0 became porous, while MB3 maintained a more preserved surface morphology, with a Young’s modulus 1.6-fold greater than that of MB0 (Fig. 2B).

Membrane integrity was assessed by SYTOX Green uptake (*19*), which measures membrane permeability. Without formaldehyde, MB0 and MB3 showed similarly low permeability (3.0 and 6.3%, respectively) (Fig. 2C). After 6 hours in 6 mM formaldehyde, permeability in MB0 surged to 30.5%, whereas MB3 exhibited substantially reduced membrane permeability (7.3%) (Fig. 2C). The increase in permeability was nearly 28-fold larger in MB0 than in MB3 (Fig. 2C).

We then asked whether morphological remodeling also protects against genotoxic damage. Formaldehyde is known to induce DPCs (*5*). Following overnight exposure to 6 mM formaldehyde, the DPC coefficient (*20*) increased to 86% in MB0 (82% over untreated control) but only to 62% in MB3 (57% over control) (Fig. 2D). Thus, the MreB(A53R)-engineered strain exhibited reduced formaldehyde-induced DPC accumulation.

Formaldehyde also causes protein misfolding and aggregation (*6*). After overnight exposure to 6 mM formaldehyde, soluble protein concentration in MB0 dropped by 57% (from 0.61 to 0.26 mg/ml) (Fig. 2E), whereas MB3 showed only a 17% decrease (from 0.58 to 0.47 mg/ml) (Fig. 2E). In addition, formaldehyde-induced ROS levels (*7*) were 21% lower in MB3 than in MB0 (Fig. 2F), consistent with reduced cellular damage under formaldehyde stress.

To explore the structural basis for these effects, we modeled wild-type and A53R MreB. The A53R substitution, located on a surface β sheet, did not alter the overall fold [root mean square deviation (RMSD) = 0.091 Å] (fig. S1A) or disrupt key hydrogen bonds (fig. S1B),but introduced a positive charge and increased side-chain steric hindrance. Molecular docking analysis suggested a potential alteration of the local adenosine 5′-triphosphate (ATP)–binding environment caused by the A53R substitution (ΔG_bind from −10.13 to −17.36 kcal·mol^−1^) (fig. S1C), and these structural predictions provide a possible molecular explanation for the altered cellular phenotype, although direct measurements of MreB polymerization dynamics and ATP-binding properties will be required to further establish this mechanism. Under formaldehyde stress, the A53R-expressing strain (MB3) showed increased peptidoglycan thickness, higher cell stiffness, better membrane integrity, and reduced DPCs, protein aggregation, and ROS level. Collectively, these data indicate that a single–amino acid substitution in MreB remodels cellular architecture and improves envelope-associated resistance to formaldehyde-induced damage. These observations suggest that changes in bacterial architecture and envelope properties may represent an additional layer of physiological adaptation that influences cellular stress responses.

### Chemical perturbation with A22 separates acute morphological protection from sustained metabolic adaptation

To determine how MreB-associated morphological states contribute to formaldehyde tolerance—and to dissect the causal hierarchy between shape change and metabolic rewiring—we used A22, a well-characterized MreB-specific chemical perturbant that disrupts MreB-associated cytoskeletal dynamics (*11*). We treated the control strain MB0 and the shape-altered strain MB3 with either DMSO (mock) or A22 (8 mg/liter) and cultivated them in the presence of 1 mM formaldehyde.

SEM confirmed that MB0 + mock cells retained the typical long-rod morphology (mean length of 1.77 μm, aspect ratio of 3.19) (Fig. 2G) (fig. S2). A22 treatment induced a morphological shift toward shorter and rounder cells (mean length of 1.53 μm, aspect ratio of 1.8) (Fig. 2G and fig. S2), partially recapitulating the morphology associated with the A53R allele. MB3 + mock cells already exhibited a short-rod morphology, closely resembling A22-treated wild-type cells (Fig. 2G and fig. S2). Addition of A22 to MB3 further altered the cells toward a spherical shape (Fig. 2G and fig. S2), indicating that the A53R allele alters the cellular response to MreB perturbation but does not eliminate sensitivity to A22.

We next assessed formaldehyde tolerance by measuring growth (ΔOD_500_) over 24 hours. At the 21-hour time point, MB0 + mock grew poorly (ΔOD_500_ = 0.14) (Fig. 2H). Notably, A22 treatment alone improved tolerance of wild-type cells nearly fivefold (MB0 + A22, ΔOD_500_ = 0.69) (Fig. 2H), suggesting that acute MreB perturbation–associated morphological remodeling can provide rapid protection against formaldehyde stress. MB3 + mock showed the highest tolerance (ΔOD_500_ = 0.83), a 5.9-fold improvement over MB0 + mock (Fig. 2H). However, cotreatment of MB3 with A22 fully abolished this benefit (ΔOD_500_ = 0.02), even below the level of A22-treated wild-type cells (Fig. 2H). These results indicate that the enhanced formaldehyde tolerance associated with the A53R background depends on the continued presence of functional MreB-associated cellular processes—it is not a permanent state locked by the mutation but rather a continuously maintained physiological condition.

Consistent with these growth phenotypes, membrane integrity, DPC accumulation, and ROS levels all followed the same pattern. MB0 + mock showed high DPC coefficient of 26.9% and elevated ROS level. A22 treatment reduced these stress markers in MB0, and the MB3 + mock strain performed even better. However, adding A22 to MB3 substantially reduced these protective effects, increasing DPC coefficient to 24.6% (Fig. 2I) and ROS levels by 28% relative to MB3 + mock (Fig. 2J).

Together, these chemical-genetic experiments establish two key points. First, acute chemical perturbation of MreB induces a morphology-associated protective response that improves tolerance to formaldehyde stress (tier 1). Second, the enhanced stress tolerance associated with the A53R strain requires an intact MreB-dependent cellular state, as it is fully abolished by A22. Together, these chemical-genetic experiments support a model in which MreB-associated morphological remodeling provides a rapid envelope-associated protective response against acute formaldehyde stress, while sustained adaptation requires longer-term cellular remodeling. Although chemical perturbation approaches may involve indirect effects, the consistency between A22-induced phenotypes and those observed in the genetically engineered MreB(A53R) strain supports the involvement of MreB-associated morphological regulation.

### MreB(A53R)-associated cellular remodeling enhances methanol assimilation beyond acute morphological protection

Because the MreB A53R strain (MB3) exhibited enhanced tolerance to formaldehyde—a central intermediate in C1 metabolism (Fig. 1A) (*21*)—we asked whether this morphological remodeling also improves assimilation of C1 substrates. We focused on methanol as a model substrate, given its relevance to methylotrophy (*22*) and its compatibility with engineered *E. coli*.

To enable methanol assimilation, we introduced the ribulose monophosphate (RuMP) pathway (*23*) into a strain background lacking endogenous formaldehyde detoxification (*frmA*) and pentose phosphate pathway (PPP) genes (*rpiAB* and *cyaA*) (Fig. 3A) (*8*). The resulting control strain (MB6) and the shape-altered strain (MB7, carrying MreB A53R) were cultivated in Mops minimal medium with 50 mM xylose, 2% casamino acids, and varying methanol concentrations.

**Fig. 3.**
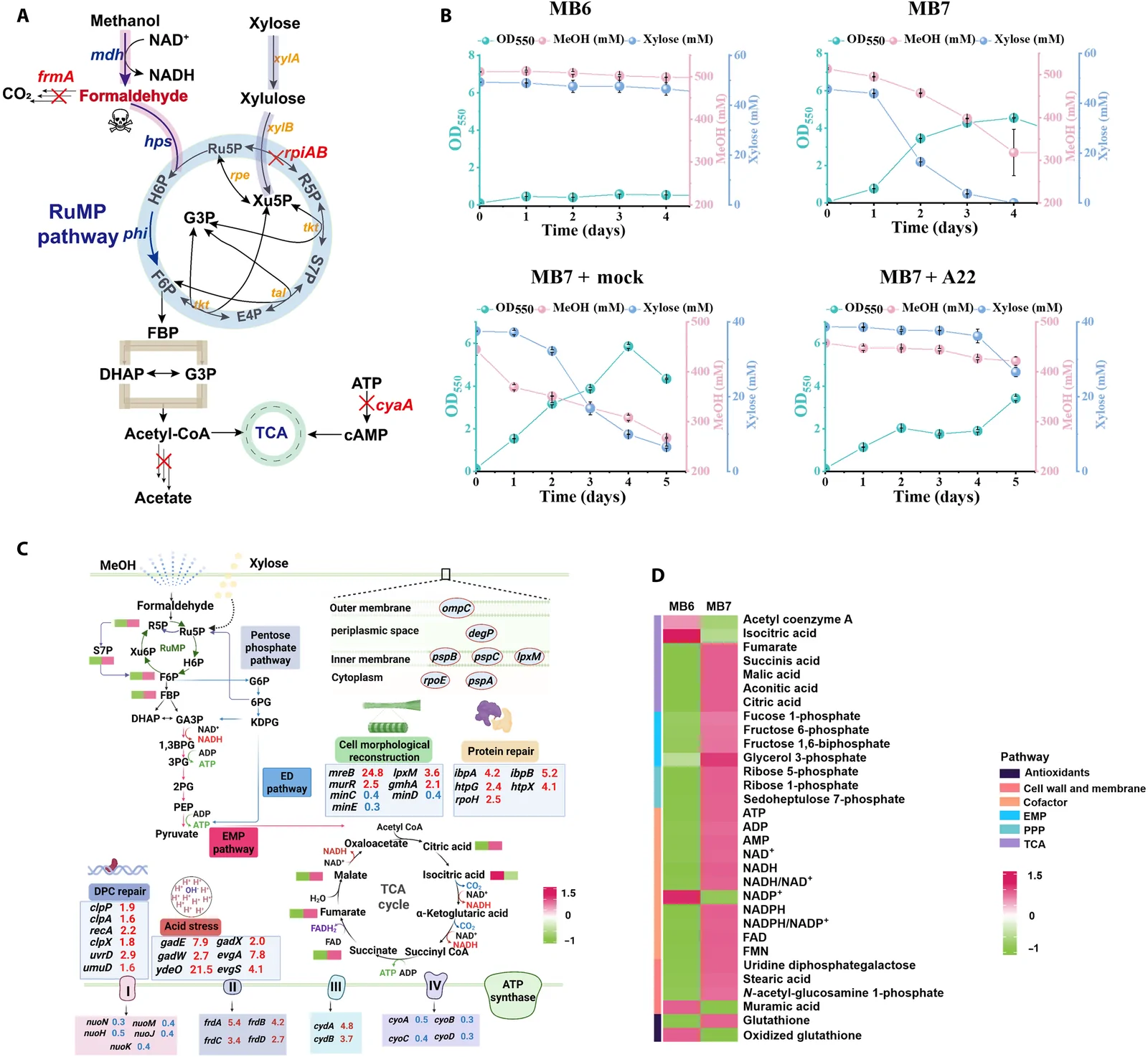
MreB(A53R) enhances methanol assimilation in *E. coli*. (**A**) Schematic illustration of the methanol assimilation-linked central carbon metabolic pathway. Gene knockouts are marked with red “×” symbols. Green segments represent heterologously introduced RuMP pathway genes, including methanol dehydrogenase (*Mdh*) from *Cupriavidus necator*, and 3-hexulose-6-phosphate synthase (*Hps*) and 6-phospho-3-hexulose isomerase (*Phi*) from *B. methanolicus*. Blue segments denote endogenous genes. FrmA, S-(hydroxymethyl)glutathione dehydrogenase; RpiAB, ribose-5-phosphate isomerase A/allose-6-phosphate isomerase; CyaA, adenylate cyclase; XylA, xylose isomerase; XylB, xylulokinase; Rpe, ribulose-phosphate 3-epimerase; Tkt, transketolase; Tal, transaldolase. (**B**) Growth kinetics and substrate utilization profiles of strains MB6, MB7, MB7 + mock, and MB7 + A22 (8 mg/liter). Cells were cultured in 10 ml of Mops medium containing 450 mM methanol and 50 mM xylose in 50-ml conical tubes. (**C**) MA plot showing global transcriptomic differences between MB6 and MB7. Red/blue numbers indicate fold change (up/down) with adjusted *P* < 0.05. (**D**) Key metabolite levels (liquid chromatography–mass spectrometry) after 48 hours of cultivation. Data are mean ± SD (*n* = 3). Statistical significance between MB6 and MB7 was determined by two-tailed unpaired Student’s *t* test. MeOH, methanol; ADP, adenosine diphosphate.

Neither strain grew detectably without methanol under these conditions (with xylose and casamino acids present), indicating that methanol is essential for growth in this engineered background. At 450 mM methanol, MB6 reached a maximum OD_550_ of 0.5 and consumed only 13 mM methanol and 3 mM xylose over 4 days (Fig. 3B), consistent with previously reported RuMP-based strains. In stark contrast, MB7 grew rapidly, reaching OD_500_ 4.6, and consumed approximately 200 mM methanol and 50 mM xylose—a 9.2-fold, 15-fold, and 17-fold improvement, respectively (Fig. 3B). At 900 mM methanol, MB6 barely grew, whereas MB7 reached OD_500_ 1.87 and consumed 142 mM methanol and 38 mM xylose (Fig. 3B) (after correction for volatilization). ^13^C-methanol labeling (*24*) confirmed the incorporation of methanol-derived carbon into Embden-Meyerhof-Parnas Pathway, PPP, and tricarboxylic acid (TCA) cycle metabolites (fig. S3). These results demonstrate that the MreB(A53R)-engineered strain exhibits enhanced methanol assimilation capacity.

We then asked whether acute pharmacological induction of the short-rod morphology alone, using the MreB inhibitor A22, is sufficient to enhance methanol assimilation—as it was for formaldehyde tolerance. Wild-type cells (MB6 background) treated with A22 (MB6 + A22) showed no improvement in growth or methanol consumption compared to mock-treated controls; both groups consumed negligible methanol over 4 days. This result reveals a functional distinction between rapid morphological adaptation and sustained metabolic remodeling: although acute MreB perturbation–associated morphological changes improved formaldehyde tolerance, they were insufficient to support efficient methanol assimilation.

In MB7 (the A53R strain), A22 treatment greatly impaired methanol consumption (from ∼178 to 35 mM over 5 days) (Fig. 3B), consistent with inhibition of MreB-associated cellular processes. However, unlike the complete collapse of formaldehyde tolerance observed in MB3 + A22—a phenotype that depends acutely on MreB-mediated physical defense (tier 1)—MB7 + A22 retained residual but notable methanol assimilation capacity (35 mM consumed, still exceeding the control MB6’s 13 mM). This residual activity may reflect the persistence of previously established metabolic adaptations in the A53R background after acute MreB perturbation. These adaptations, including altered redox balance and carbon flux distribution observed in the A53R strain, may support continued but reduced C1 assimilation. In contrast, the immediate physical protection (tier 1) vanishes instantly upon A22 addition, explaining the stark difference in sensitivity between the two assays. Together, these results support a two-stage adaptive model: a rapid morphology-associated protective response (tier 1) and a sustained cellular adaptation associated with transcriptional and metabolic remodeling (tier 2).

### Multiomics reveals cellular metabolic and redox remodeling associated with MreB(A53R)

To understand how morphological remodeling enhances methanol assimilation, we performed integrated transcriptomic and metabolomic analyses on the shape-altered strain MB7 and the control MB6.

### Envelope and stress response

The short-rod strain showed increased expression of genes involved in cell wall remodeling (*mepS*, 9.7-fold) (*25*) and outer membrane stabilization (*lpxM*, 3.6-fold) (*26*) (Fig. 3C), consistent with the observed increase in peptidoglycan thickness. Envelope stress-responsive genes were strongly induced: The periplasmic protease/chaperone (*degP*, 4.1-fold) (*27*), the membrane integrity regulator (*pspA*, 6.5-fold) (*28*), and (the *pspBCDE* operon, 2.4- to 3.6-fold) (*28*) were all up-regulated (Fig. 3C), indicating an active envelope stress response. The outer membrane porin *ompC* (*29*) was also increased (2.2-fold) (Fig. 3C).

#### DNA repair and protein quality control

Transcripts involved in DPC repair (*recA*, 2.2-fold; *uvrD*, 2.9-fold) (*30*) and protein homeostasis (*ibpA*, 4.2-fold; *ibpB*, 5.2-fold; *htpG*, 2.4-fold; *htpX*, 4.1-fold) (*31*, *32*) were elevated (Fig. 3C). The ROS-scavenging enzyme *sodB* (*7*) increased sixfold (Fig. 3C). Thus, the MreB(A53R)-associated cellular state is accompanied by coordinated activation of multiple stress defense programs.

#### Carbon flux redirection

The global carbon regulator *cra* was up-regulated 2.4-fold. Most notably, *zwf* (encoding glucose-6-phosphate dehydrogenase) increased 2325-fold, and *edd* (Entner-Doudoroff pathway) increased 330-fold (Fig. 3C), indicating transcriptional reinforcement of PPP and ED pathway capacity. Consistent with this, metabolomics showed accumulation of PPP intermediates (ribose-5-phosphate and sedoheptulose-7-phosphate), elevated NADPH, and an increased NADPH/NADP^+^ ratio (Fig. 3D).

#### Energy metabolism remodeling

Expression of electron transport chain components shifted: Complexes I and IV were down-regulated, complexes II and III were up-regulated, and complex V remained stable (Fig. 3C). This reconfiguration may contribute to reduced oxidative stress by altering electron transport chain balance (*33*). Metabolomics confirmed lower levels of TCA cycle intermediates, reduced glutathione increased while oxidized glutathione decreased, and ATP and NADH levels were elevated (Fig. 3D)—all consistent with a more reduced intracellular environment and lower oxidative stress.

The integrated data collectively indicate that morphological remodeling establishes a redox-optimized cellular state: Carbon flux redirected toward NADPH-generating pathways, ETC reconfigured to minimize ROS production, and stress responses preemptively activated.

### RpoE, Cra, and GadE contribute to the enhanced methanol assimilation phenotype

The multiomic analysis identified RpoE, Cra, and GadE as candidate regulators associated with the MreB(A53R)-induced adaptive state. To test whether these regulators are functionally required for the enhanced methanol assimilation of the shape-altered strain, we performed targeted genetic perturbations in the MB7 background.

RpoE (σ^E^) is a sigma factor that governs the envelope stress response, activating genes involved in periplasmic chaperones, proteases, and lipopolysaccharide biosynthesis (*34*). Cra is a global transcription factor that directs carbon flux, activating the PPP and gluconeogenesis while repressing glycolysis (*35*). GadE is the central activator of the glutamate-dependent acid resistance system (*36*). Transcriptomic data confirmed that all three were up-regulated in MB7 compared with the control MB6 (*rpoE* 3.4-fold, *cra* 2.4-fold, *gadE* 7.9-fold), suggesting a coordinated response.

Because *rpoE* and *cra* are essential in *E. coli*, we used CRISPR interference (CRISPRi) (*37*) to conditionally suppress their expression. *gadE* was directly deleted. The resulting strains—MB7-*rpoE*i, MB7-*cra*i, and MB7-Δ*gadE*—were cultivated in Mops medium with 450 mM methanol and 50 mM xylose, alongside the parent MB7 and the control MB6.

MB7 grew robustly, consuming ∼200 mM methanol over 4 days. In contrast, all three perturbed strains showed severely impaired growth and methanol consumption, with final OD_500_ and methanol levels comparable to those of MB6 (fig. S4). Thus, each of these regulators contributes to maintaining the enhanced phenotype observed in the MreB(A53R) background.

RpoE is required for membrane stress adaptation and methanol utilization. Knockdown of *rpoE* reduced expression of envelope stress-responsive genes (*degP*, *lpxM*, and *pspA*) (*38*) and xylose assimilation genes (*xylA*, *xylB*, and *xylE*) (Fig. 4A) and also suppressed TCA cycle activity (Fig. 4B). This indicates that RpoE-associated envelope stress adaptation contributes to maintaining metabolic efficiency in the MreB(A53R) background.

**Fig. 4.**
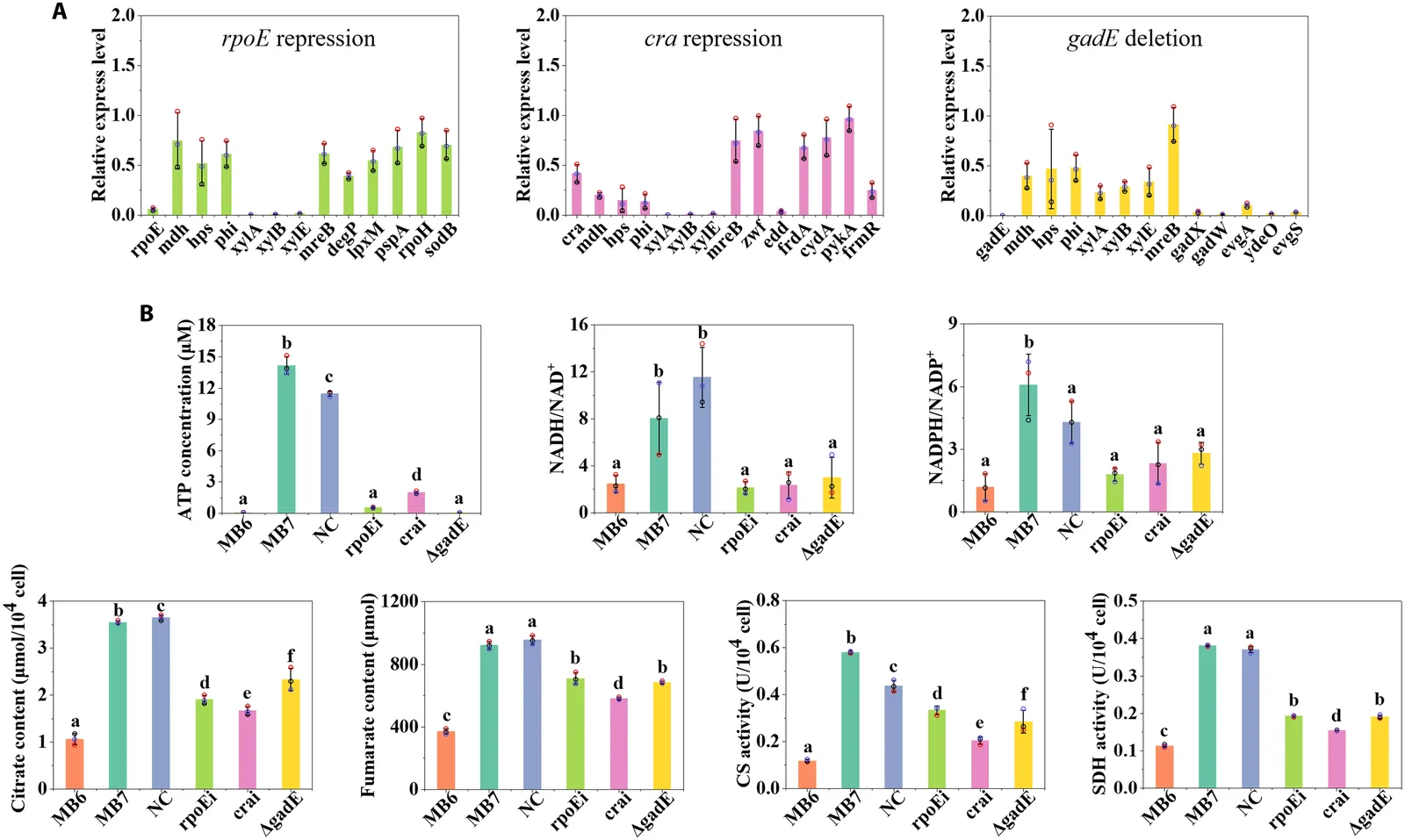
The RpoE-Cra-GadE regulatory unit is essential for enhanced methanol metabolism. (**A**) CRISPRi-mediated repression of *rpoE* and *cra* and deletion of *gadE*. Bar plots show relative expression levels of representative target genes, normalized to a nontargeting negative control (NC). (**B**) Metabolic consequences of transcriptional factor repression or knockout. Bar plots show intracellular ATP levels; NADH/NAD^+^ and NADPH/NADP^+^ ratios; citrate and fumarate concentrations; and activities of citrate synthase (CS) and succinate dehydrogenase (SDH) in NC, *rpoE* i, *cra* i, and Δ*gadE* strains. Different lowercase letters (a to e) indicate significant differences between groups (*P* < 0.05, one-way ANOVA followed by Tukey’s HSD post hoc test). Groups sharing the same letter are not significantly different. Data are mean ± SD (*n* = 3).

Cra is essential for carbon flux redistribution. *Cra* knockdown down-regulated RuMP pathway, ED pathway, and TCA-related genes (*mdh*, *hps*, *edd*, and *frdA*) (Fig. 4A), reduced ATP, NADH/nicotinamide adenine dinucleotide (NAD^+^), and NADPH/NADP^+^ ratios (Fig. 4B), lowered citrate and fumarate levels, and diminished citrate synthase and succinate dehydrogenase activities (Fig. 4B). Thus, Cra-associated regulation of carbon allocation toward PPP and energy-generating pathways contributes substantially to methanol assimilation.

GadE contributes to redox homeostasis beyond acid resistance. Deletion of *gadE* not only repressed acid resistance genes (*gadX*, *gadW*, *evgA*, and *ydeO*) (Fig. 4A) (*36*) but also impaired methanol and xylose utilization, reduced NADPH/NADP^+^ ratio and citrate synthase activity (Fig. 4B), and disrupted redox balance. This suggests that GadE appears to contribute to intracellular redox homeostasis under methanol-assimilation conditions. Collectively, these genetic perturbations demonstrate that RpoE, Cra, and GadE are key regulatory components required for maintaining the enhanced methanol assimilation phenotype associated with MreB-mediated morphological remodeling. In this model, the MreB(A53R)-associated cellular state is accompanied by coordinated activation of envelope stress responses, carbon redistribution programs, and redox regulatory pathways. RpoE, Cra, and GadE represent interconnected regulatory components that collectively contribute to maintaining the enhanced methanol assimilation phenotype. Although other envelope stress pathways, such as the Rcs system, may also respond to altered cell envelope states, our genetic perturbation data identify RpoE, Cra, and GadE as important contributors to the adaptive phenotype observed in the MreB(A53R) background. The resulting coordinated reduction in ROS generation and optimization of carbon and energy metabolism establishes a redox-optimized state that sustains high C1 flux under formaldehyde-induced stress.

### Stability and scalability of this chemical genetic strategy

We next assessed long-term stability by serial subcultivation in baffled shake flasks with transfers every 24 hours for 11 rounds (Fig. 5A). MB7 maintained robust growth throughout, with final OD_500_ consistently reaching 11 to 14 in each round (Fig. 5A). Methanol and xylose were steadily consumed in every round (Fig. 5A), indicating that the strain retained its ability to co-utilize these carbon sources during prolonged cultivation. Slight fluctuations in substrate consumption occurred between rounds, but overall patterns remained largely consistent over the 11 passages. Although we did not perform a dedicated competitive fitness assay under nonstress conditions, the engineered strain showed stable growth and substrate utilization during 11 sequential passages, indicating that the MreB(A53R)-mediated remodeling was compatible with sustained cellular proliferation under the tested conditions.

**Fig. 5.**
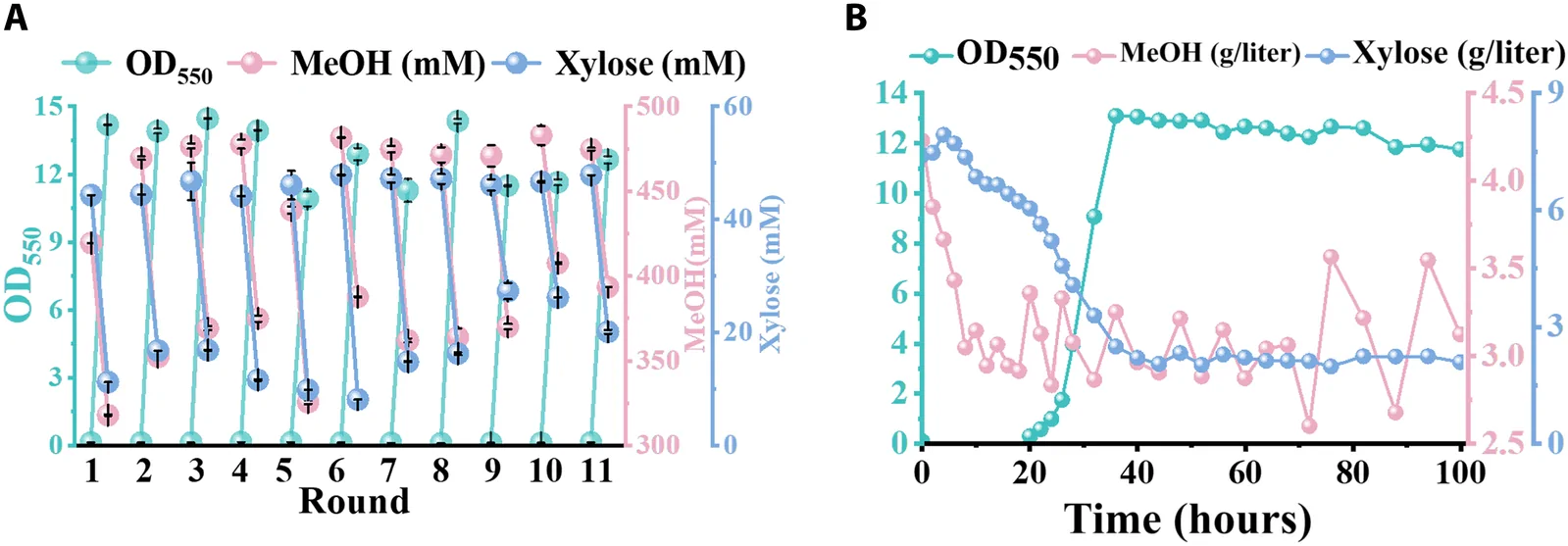
Stability and scalability of the chemical genetic system. (**A**) Long-term stability of the perturbed division phenotype. Strain MB7 was serially passaged every 24 hours for 11 rounds (11 days) in baffled flasks containing Mops medium supplemented with ∼500 mM methanol, 50 mM xylose, and 2% casamino acids. Methanol consumption and OD_550_ were measured at the end of each round. (**B**) Fed-batch fermentation of strain MB7 in a 5-liter bioreactor. Methanol and xylose concentrations were maintained at ∼100 and >10 mM, respectively, via continuous feeding. OD_550_, methanol consumption, and xylose consumption were monitored over 100 hours. Data are mean ± SD (*n* = 3).

To assess scalability, we performed fed-batch fermentation of MB7 in a 5-liter bioreactor with automatic substrate feeding to maintain xylose above 10 mM and methanol above 100 mM. After 100 hours, MB7 consumed 402 mM xylose and 1027 mM methanol (Fig. 5B). These results highlight the robust substrate utilization capacity and strong scalability of the shape-altered strain under high C1 feeding conditions.

### The chemical genetic strategy extends to other C1 substrates and a native methylotroph

We next investigated whether the shape-altered strain could also improve assimilation of additional C1 compounds. Given the substantial metabolic overlap among formaldehyde, methane, and methanol (*39*), the mechanisms identified here likely extend to formaldehyde. We also tested the formyl-CoA elongation (FORCE) pathway (*40*), an orthogonal framework that enables formaldehyde utilization and produces glycolate, a valuable chemical for pharmaceuticals and biodegradable materials.

The FORCE pathway was implemented by expressing four key enzymes: 2-hydroxyacyl-CoA-lyase (HACL) from *Chloroflexi bacterium*, acyl-CoA reductases (ACRs) from *Listeria monocytogenes* and *Salmonella typhimurium* (EutE), and glycolaldehyde dehydrogenase (GldA) from *E. coli*, distributed across two plasmids (pET-*acr-eutE-gldA* and pCDF-*hacl*) (Fig. 6A) (*40*). Transformation with the production plasmids into the shape-altered strain MB5 and the control MB4 yielded MB19 and MB18, respectively. Within 24 hours, MB19 produced 1581 μM OD^−1^ of glycolate, which is 4.6-fold higher than the 343 μM OD^−1^ produced by MB18 (Fig. 6A), demonstrating that morphological remodeling markedly enhances even fully synthetic C1-fixing pathways.

**Fig. 6.**
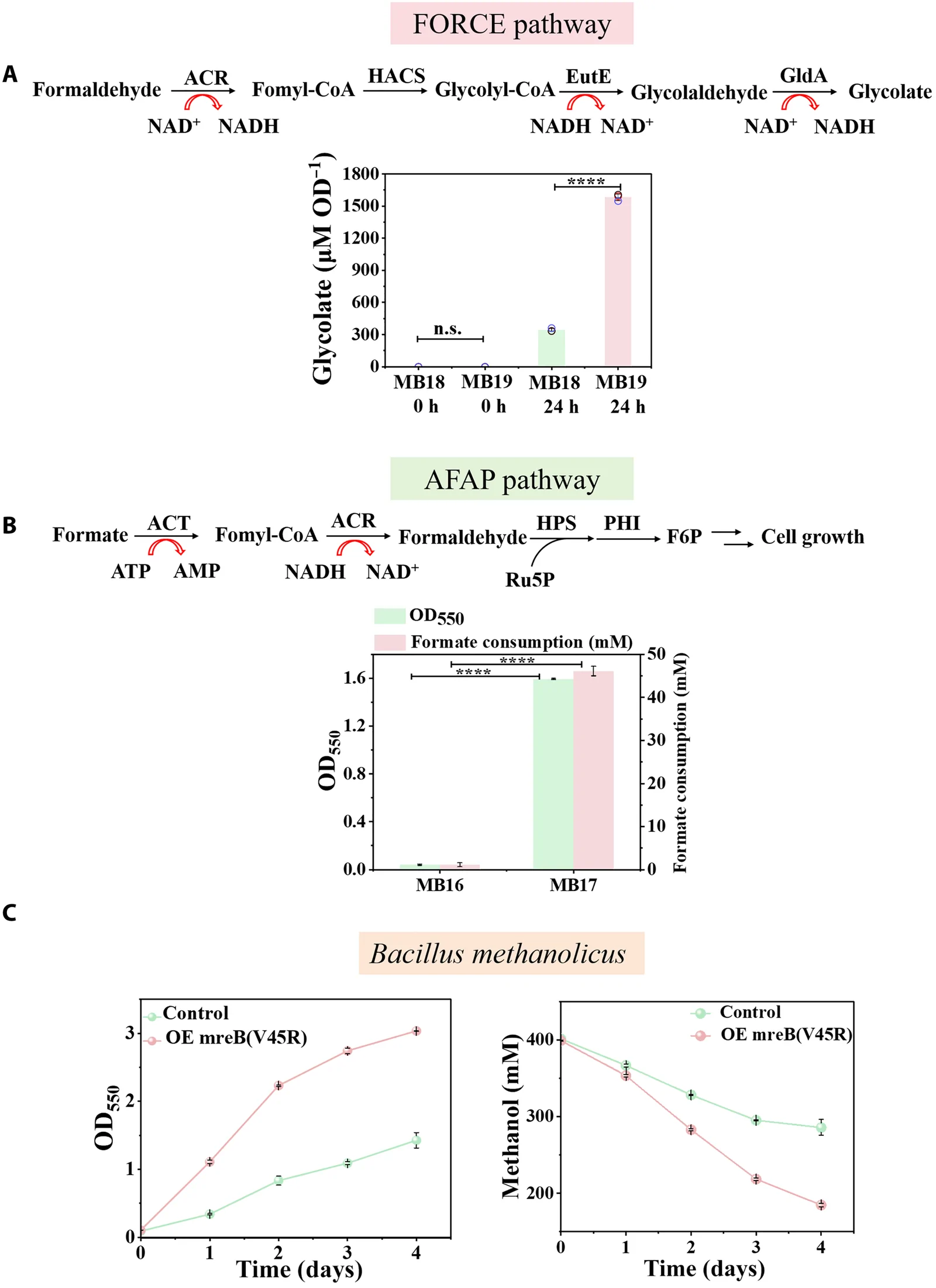
The chemical-genetic system enhances utilization of other C1 substrates in *E. coli* and methanol utilization in the native methylotroph *B. methanolicus*. (**A**) Glycolate production from the FORCE pathway by control strain MB18 and chemical-genetic strain MB19. Cells were cultured in Mops medium containing ∼20 mM formaldehyde. “n.s.” means no significance, and “****” means *P* ≤ 0.0001, unpaired two-tailed Student’s *t* test or appropriate post hoc comparison. (**B**) Formate assimilation via a synthetic AFAP pathway. Formate is converted to formaldehyde by acyl-CoA transferase (ACT) (ACS from *S. typhimurium*) and ACR (ACDH2 from *L. monocytogenes*), followed by catalysis via HPS and PHI to enter glycolysis. Growth (OD_550_) and formate consumption of control strain MB16 and chemical-genetic strain MB17 were measured over 48 hours in Mops medium containing 50 mM formate. “****” means *P* ≤ 0.0001, unpaired two-tailed Student’s *t* test or appropriate post hoc comparison. (**C**) Growth curve (OD_550_) and methanol consumption of wild-type MGA3 and a strain overexpressing MreB (V45R) was assessed in MVcMY minimal medium containing 400 mM methanol. Cultures were incubated at 50°C in a 50-ml conical tube.

We further assessed formate assimilation. As *E. coli* lacks efficient native formate pathways, we engineered an artificial formate assimilation pathway (AFAP) in which formate is first converted to formaldehyde via acyl-CoA transferase (ACT) and ACR (Fig. 6B) (*41*), followed by HPS/PHI-catalyzed entry into glycolysis. The pathway was assembled on plasmid pCDF-P1-*StACS*-*LmACDH2*-P2-*hps*-*phi*, expressing ACT from *S. typhimurium*, ACR from *L. monocytogenes*, and HPS/PHI from *B. methanolicus*. In the shape-altered strain (MB17), biomass increased sharply (OD_500_ from 0.1 to 1.6) within 48 hours (Fig. 6B), accompanied by consumption of 46 mM formate—a 45-fold enhancement over the MB16 control (1 mM) (Fig. 6B) (*P* < 0.01).

To examine whether MreB-associated morphological engineering could be extended to a native methylotrophic chassis, we investigated *B. methanolicus* MGA3 (*42*). Sequence alignment of MreB from MGA3 (BMMGA3_12545) and *E. coli* MG1655 (b3251) revealed that residue V45 in *B. methanolicus* corresponds to A53 in *E. coli* (fig. S5). Both valine and alanine are nonpolar, hydrophobic residues; on the basis of the conserved position and the introduction of a positively charged residue at this site, we generated the V45R variant to examine whether analogous MreB perturbation could produce a comparable cellular phenotype in *B. methanolicus*. We cloned MreB (V45R) gene into the pTCas9 plasmid (with Cas9 removed) under a mannitol-inducible promoter (PmtlA) (*43*). The pTCas9-PmtlA-MreB (V45R) plasmid was introduced into MGA3 to generate the *mreB* (V45R)-overexpressing strain [OE *mreB*(V45R)]. The empty vector pTCas9-PmtlA served as the control. In parallel, OE *mreB*(WT) strain was constructed under the same promoter to distinguish the effect of the specific MreB variant from a general consequence of increased MreB abundance. SEM analysis showed that overexpression of wild-type MreB did not cause obvious morphological changes compared with the empty vector control (fig. S7), whereas expression of *mreB*(V45R) resulted in significantly increased cell width and reduced aspect ratio (fig. S6), partially resembling the morphological shift observed for EcMreB(A53R).

We then assessed methanol assimilation. All strains were cultivated in MVcMY minimal medium (*44*) with 5 mM mannitol (inducer) and 400 mM methanol as the sole carbon source. Consistent with the morphological observations, the OE *mreB*(WT) strain showed no substantial improvement in growth or methanol consumption compared with the empty vector control. In contrast, the OE *mreB*(V45R) strain exhibited enhanced growth and methanol consumption, reaching an OD_500_ of 3.0 after 96 hours (Fig. 6C). Methanol consumption mirrored this pattern. The OE *mreB*(V45R) strain consumed 215 mM methanol, nearly twice the amount consumed by the control (115 mM) (Fig. 6C). These results indicate that engineering MreB-associated cellular remodeling can improve methanol assimilation capacity in an additional methylotrophic chassis.

## DISCUSSION

Microbial conversion of C1 compounds represents a promising sustainable biomanufacturing route, yet its large-scale implementation is persistently constrained by intrinsic physiological conflicts: C1 metabolic pathways inevitably generate formaldehyde, a highly toxic intermediate that triggers cell envelope damage, DPCs, protein misfolding and aggregation, and excessive ROS accumulation (*45*, *46*). Meanwhile, C1 assimilation imposes severe redox imbalance that mismatches the metabolic network evolved for conventional sugar catabolism (*45*, *46*). Traditional strategies to address these challenges rely on iterative metabolic pathway modification or long-term adaptive evolution, approaches that are labor intensive, strain specific, and difficult to generalize across microbial chassis. In this study, we exploited a chemical genetic toolkit consisting of an MreB gain-of-function variant (A53R) and the reversible inhibitor A22 to dissect the biological function of the bacterial actin homolog MreB beyond its well-characterized structural role. Our results suggest that MreB functions beyond its classical structural role as a physiological state integrator, whereby alterations in cytoskeletal organization and cell envelope architecture are associated with coordinated stress responses, metabolic remodeling, and improved C1 adaptation, revealing an unrecognized regulatory layer in bacterial physiology.

A key finding of this work is the discovery of a two-tiered functional mechanism underlying MreB-mediated stress resistance and metabolic reprogramming, which neatly decouples immediate physical protection from long-term adaptive metabolism. The first tier is a rapid, MreB-dependent physical defense response. Acute treatment with A22 alone alters cell morphology and recapitulates formaldehyde tolerance in wild-type cells, demonstrating that reshaping cells into short rods and reinforcing cell envelope structure—including thickened peptidoglycan, elevated cell stiffness, and improved membrane integrity—are sufficient to shield cells from acute formaldehyde-induced damage. This physical barrier functions as a fast-acting defense line that operates independently of de novo transcription, enabling bacteria to cope with sudden C1-related stress. The second tier is a chronic, transcription-dependent metabolic adaptation that cannot be triggered by short-term A22 intervention. The MreB A53R mutation stabilizes MreB filaments and sustains altered cytoskeletal states, which is associated with activation of RpoE, Cra, and GadE regulatory components. This regulatory network comprehensively rewires central metabolism: It remodels the electron transport chain to minimize ROS leakage, redirects carbon flux toward NADPH-generating pentose phosphate and Entner-Doudoroff pathways, and optimizes intracellular redox poise. Unlike the transient morphological change induced by A22, this metabolic adaptation may persist beyond acute MreB perturbation, allowing partial maintenance of C1 assimilation capacity after transient inhibition; even after MreB activity is inhibited, the established transcriptional and metabolic states persist for a period of time, retaining partial C1 assimilation capacity. Our findings indicate that morphological remodeling and metabolic adaptation represent interconnected but distinguishable physiological processes. Changes in cell geometry, envelope organization, surface-to-volume ratio, and membrane properties may indirectly influence nutrient transport, energy allocation, and stress perception. However, efficient C1 assimilation requires additional transcriptional and metabolic remodeling beyond morphological alteration alone, as acute A22-induced shape changes improved formaldehyde tolerance but were insufficient to enhance methanol assimilation. This two-tiered model explains how bacteria integrate rapid physical defense and sustained metabolic adaptation to survive and thrive under C1 stress, and it provides a general framework for understanding cytoskeleton-stress-metabolism cross-talk in prokaryotes.

Our multiomic analysis and targeted genetic perturbations identify RpoE, Cra, and GadE as key regulatory components associated with the MreB(A53R)-induced adaptive state in MreB-driven C1 metabolism remodeling. As the master regulator of the envelope stress response, RpoE senses cell envelope changes triggered by MreB conformational shifts and initiates downstream stress signaling. The global carbon regulator Cra then reroutes carbon flux to prioritize NADPH production, a cofactor critically required for formaldehyde detoxification and C1 substrate assimilation. GadE, originally known for acid resistance, further maintains intracellular redox homeostasis to stabilize the remodeled metabolic network. The coordinated action of these three transcription factors builds a robust regulatory circuit that links cytoskeletal perturbation to whole-cell metabolic reprogramming. While we have defined the downstream transcriptional regulator, the molecular mechanism by which MreB filament dynamics and cell shape changes transmit signals to activate RpoE remains to be fully elucidated. We hypothesize that altered membrane tension, peptidoglycan remodeling, or changes in membrane-associated protein distribution may serve as intermediate signal mediators. In addition to the RpoE-associated envelope stress response described here, other envelope surveillance systems may potentially contribute to sensing MreB-associated structural alterations. For example, the Rcs phosphorelay has been implicated in responding to changes in cell envelope organization caused by MreB perturbation and mechanical stress. Although our current data do not directly evaluate Rcs pathway activity, future investigation of potential cross-talk between MreB, RpoE, Rcs, and other envelope stress networks will provide a more comprehensive understanding of cytoskeleton-mediated signaling in bacteria. This open question warrants further investigation in future work, which will deepen our mechanistic understanding of cytoskeleton-to-signal transduction in bacteria. Although these regulators are genetically required for maintaining the enhanced phenotype, whether they operate through a strict linear hierarchy or through partially interconnected regulatory modules remains unresolved.

From a chemical biology perspective, this study validates MreB as a druggable and manipulable target for cellular reprogramming. The two orthogonal tools we used produce distinct physiological outcomes: The small-molecule inhibitor A22 induces rapid morphological changes and acute stress tolerance, which can be applied as a chemical preconditioning agent to protect microbial cells during C1 bioprocessing. In contrast, the heritable MreB A53R mutation generates a stable, long-term metabolic phenotype suitable for constructing robust industrial microbial chassis. This dual-mode regulation highlights the unique advantages of chemical genetics: It enables us to selectively tune protein function to achieve either transient physiological modulation or stable strain engineering. Given the conservation of MreB among many rod-shaped bacteria, this strategy may have broader applicability beyond *E. coli*. We provided initial evidence for transferability by introducing MreB(V45R) into the native methylotroph *B. methanolicus*, which reproduced improved methanol utilization. Successful engineering of additional C1 assimilation pathways— including the FORCE pathway for glycolate synthesis and the AFAP— further proves that MreB modulation is a universal strategy to enhance C1 bioconversion, regardless of the specific metabolic routes adopted.

We also verified the practical potential of this strategy for industrial application via serial passaging and 5-liter fed-batch fermentation. The engineered strain maintained stable growth and high C1 substrate consumption during long-term cultivation and large-scale fermentation, eliminating concerns about phenotypic instability, a common problem in metabolically engineered strains. These results lay a solid foundation for the industrial translation of this technology. For future industrial development, combining MreB engineering with classic metabolic pathway optimization may yield even higher C1 conversion efficiency and target product titer. In addition, since MreB is essential for bacterial survival and morphology maintenance, its inhibitors have long been considered promising antibacterial agents. As with other morphology-based engineering strategies, potential physiological trade-offs associated with MreB modification should be carefully considered. Because MreB coordinates cell shape, cell wall synthesis, and chromosome dynamics, excessive alteration of MreB activity may impose fitness costs under nonstress conditions. In our study, the A53R variant improved C1 stress adaptation without eliminating cellular growth capacity under the tested conditions; however, the long-term evolutionary stability, ecological fitness, and performance across different growth environments require further evaluation before industrial implementation. Our findings expand the application scope of MreB-targeted compounds: Beyond antimicrobial therapy, MreB modulators can be repurposed as tools for microbial metabolic engineering.

In summary, this work redefines the biological role of the prokaryotic cytoskeletal protein MreB, shifting our understanding from a purely structural element to a high-order signaling node that interconnects cell morphology, stress response, and global metabolism. The two-tiered working mechanism we uncovered reveals a sophisticated adaptive strategy evolved in bacteria to cope with C1-related stress. Beyond fundamental microbiology advances, our chemical genetic strategy provides a promising framework for engineering stress-resilient microbial cell factories for C1 bioconversion. We anticipate that this cytoskeleton-based engineering concept may be explored in additional bacterial chassis and biomanufacturing contexts, opening unexpected avenues at the intersection of chemical genetics, bacterial physiology, and synthetic biology.

## MATERIALS AND METHODS

### Strains and plasmids

All bacterial strains and plasmids are listed in tables S1 and S2. *E. coli* MG1655 was used as the parental strain for tolerance evaluation, and MG1655(DE3) served as the chassis strain for C1 substrate utilization and product biosynthesis.

### Strain construction

Chromosomal integration of *mreB* at the *pta* locus and deletion of *frmA*, *pta*, *rpiAB*, and *gadE* were performed using a CRISPR-based approach. Briefly, the target strain harboring pEcCas was induced with l-arabinose and made electrocompetent. The pEcgRNA plasmid targeting the desired locus, together with donor DNA, was cotransformed. Positive transformants were selected on kanamycin and spectinomycin. The pEcgRNA plasmid was cured with l-rhamnose, and pEcCas was eliminated by transfer to antibiotic-free medium.

Deletion of *cyaA* was performed using one-step FLP-FRT recombination. The target strain carrying pKD46 was induced with l-arabinose and made electrocompetent. An FRT-Kan-FRT cassette with flanking homologous arms was electroporated, and positive colonies were selected on ampicillin. pKD46 was cured by overnight cultivation at 42°C without antibiotics.

Deletion of the native *mreB* gene was achieved via *cat-sacB* two-step allelic exchange. First, a *cat-sacB* cassette with homologous arms was integrated at the *mreB* locus, with selection on chloramphenicol. Second, sucrose counter-selection promoted excision of the cassette, generating the deletion mutant, which was verified by polymerase chain reaction (PCR) and sequencing.

CRISPRi used a dual-plasmid system (pdCas9 and pEcgRNA). pdCas9 expresses catalytically inactive *dCas9* under an anhydrotetracycline-inducible *Ptet* promoter. Guide RNAs, each with a 20-nt spacer targeting the nontemplate strand near the transcription start site, were constitutively expressed from the *P_J23119* promoter on pEcgRNA.

### Growth conditions

All MG1655-derived strains were subjected to formaldehyde stress and subsequent analyses, including tolerance assays, DPC coefficient, intracellular ROS, supernatant protein concentration, and membrane rigidity. Unless otherwise stated, experiments were performed in Mops minimal medium with 2% (w/v) glucose.

Different media were used as follows: methanol assimilation (Mops with various methanol concentrations, 50 mM xylose, and 2% casamino acids); ^13^C-methanol flux analysis (100 mM ^13^C-methanol, 50 mM xylose, and 2% casamino acids); transcriptomic/metabolomic analyses (450 mM methanol, 50 mM xylose, and 2% casamino acids); formate assimilation (50 mM formate, 50 mM xylose, and 2% casamino acids); product biosynthesis (450 mM methanol, 50 mM xylose, and 2% casamino acids); fed-batch fermentation (200 mM methanol, 50 mM xylose, and 2% casamino acids).

### AFM and SEM

AFM was used to analyze cell morphology and rigidity. Cells fixed on mica were imaged in tapping mode using a silicon cantilever (tip radius, ∼10 nm). Force-distance curves were obtained to measure elastic modulus, and data were analyzed with NanoScope analysis software.

For SEM, cells cultured for 24 hours were harvested, washed with phosphate-buffered saline (PBS), and fixed overnight in 2.5% (v/v) glutaraldehyde. After three washes with 0.1 M PBS, samples were postfixed for 1.5 hours and dehydrated in a graded ethanol series (30 to 100%). Drying was performed with a CO_2_ critical-point dryer, followed by gold sputter coating and examination.

### Continuous kinetic assays for formaldehyde tolerance

Cells from glycerol stocks were revived on agar plates. Single colonies were inoculated into Mops with 2% glucose and grown at 37°C, 220 rpm until OD_500_ = 1.5 to 2.0. Cultures were diluted to OD_500_ = 0.1 in fresh medium containing various formaldehyde concentrations, and growth was monitored for 24 hours using a microplate reader in kinetic mode.

### Cell membrane integrity assay

Stationary-phase cultures in Mops with 2% glucose were treated with different formaldehyde concentrations overnight. Cells were harvested, washed, and resuspended in PBS to OD_500_ = 1.0, and 100 μl was mixed with 900 μl of PBS containing 1 μM SYTOX Green (15-min dark incubation). Stained cells were analyzed by flow cytometry (488-nm excitation, 610-nm emission). The percentage of PI-positive cells (compromised membrane integrity) was determined from fluorescence distribution.

### Extraction and analysis of the *E. coli* cell wall

Cells grown in LB to stationary phase were harvested (5000 rpm, 5 min), washed with sterile water, and disrupted by ultrasonication. Lysates were centrifuged (11,000 rpm, 60 min, 4°C). The pellet was resuspended in 5% SDS and boiled (100°C, 60 min) until clear. The cell wall fraction was collected (39,000 rpm, 10 min), washed twice, resuspended in water, and dropped onto mica. After air drying, the peptidoglycan layer was examined by AFM, and its thickness was measured using Nanoscope Analysis software.

### Intracellular ROS measurement

ROS levels were measured using DCFH-DA. Cells were harvested, washed twice with PBS, resuspended in PBS, and incubated with 10 μM DCFH-DA for 1 hour at 37°C in the dark. After two additional washes, fluorescence was measured at 488/525 nm (ex/em).

### DPC coefficient analysis

DPCs were quantified by KCl-SDS coprecipitation. Two milliliters of culture was centrifuged (5000*g*, 10 min), washed with PBS, and lysed in 2% SDS (65°C, 10 min). Lysates were mixed with 1 M KCl in tris-HCl (pH 7.5), incubated on ice (5 min), and centrifuged (12,000*g*, 10 min, 4°C). The supernatant (free DNA) was saved. Pellets were washed twice with buffer containing 0.1 M KCl, 0.1 mM EDTA, and 0.02 M tris-HCl (pH 7.5) and then digested with proteinase K (0.2 mg/ml, 50°C, 3 hours). After centrifugation, DPC-derived DNA in the supernatant was collected. Both free and DPC-associated DNA were quantified fluorometrically with Hoechst 33258 (365/460 nm).

### Determination of free protein in supernatant

Cells were grown in Mops with 2% glucose to OD_500_ = 1.5 to 2.0 and then treated with formaldehyde for 10 hours. After centrifugation and three PBS washes, the pellet was resuspended in 2 ml of PBS and sonicated. The supernatant was collected (12,000 rpm, 5 min, 4°C), and protein concentration was determined by Bradford assay.

### Structural analysis of EC-MreB(A53R) and EC-MreB(WT) and ATP binding energy analysis

#### Structural analysis

The amino acid sequences of the target proteins were retrieved from the National Center for Biotechnology Information database and submitted to the online AlphaFold 3 server. For both proteins, the top-ranked model (model 0) with the highest confidence score was selected for subsequent analysis. Structural visualization and comparison were performed using PyMOL. The amino acid substitution (A53R) between the two proteins was explicitly annotated. Intramolecular hydrogen bond networks were analyzed, and the RMSD between the two structures was calculated to quantify conformational differences.

#### Analysis of ATP binding energy

The amino acid sequences of the target proteins were downloaded from the UniProt database (https://uniprot.org/) and submitted to the AlphaFold 3 online server (https://deepmind.google/technologies/alphafold/alphafold-server/) for protein-protein interaction (or protein-ligand interaction) structure prediction. This process generated five interaction models. The model with the optimal ranking score (model 0) was selected for in-depth interaction interface analysis to identify key interacting amino acid residues. The predicted structures were visualized using the open-source version of PyMOL 2.6, clearly displaying the specific amino acid residues involved in protein-protein (or protein-ATP) interactions.

### Omic analyses

#### Sample preparation

Strains were grown in methanol assimilation medium for 24 hours and then supplemented with 30 mM xylose for another 24 hours. Cells were harvested, frozen in liquid nitrogen, and sent for transcriptomic and metabolomic analyses. All multiomic experiments (transcriptomics and metabolomics) were performed with three independent biological replicates per condition. Each biological replicate was derived from separately cultured cell batches, and technical duplicates were included for each biological replicate to ensure assay consistency.

#### Transcriptomics

Total RNA was extracted using a commercial kit. Ribosomal RNA was depleted, and strand-specific libraries (∼450 bp) were prepared. Libraries were pooled equimolarly, diluted to 2 nM, denatured, and sequenced on an Illumina platform (paired-end). Raw read counts were normalized using the TMM (trimmed mean of M-values) method implemented in the edgeR package, followed by log_2_ transformation with a pseudo-count of 1.

#### Metabolomics

Metabolites were extracted from 30-mg cells with 50% methanol using vortexing, freeze-thaw, and grinding. After −20°C incubation, extracts were centrifuged, dried, and reconstituted in 50% methanol containing 2-chlorophenylalanine as internal standard and then filtered (0.22 μm). QC samples were pooled. Separation used an ACQUITY UPLC HSS T3 column, and detection used a Thermo Orbitrap Exploris 120 in positive/negative ion modes. Raw data were processed with MS-DIAL. Peak areas were normalized to total ion current and further scaled by Pareto scaling before multivariate analysis.

Differentially expressed genes/metabolites were identified using a false discovery rate (FDR) < 0.05 (Benjamini-Hochberg correction) combined with an absolute log_2_ fold change ≥ 1 for transcriptomic and proteomic data. For metabolomic data, we applied VIP (variable importance in projection) ≥ 1.0 from partial least-squares discriminant analysis, together with Student’s *t* test *P* < 0.05 (FDR-corrected). Functional enrichment analyses (GO and KEGG) were performed using the hypergeometric test with an FDR < 0.05 as the significance threshold.

### Metabolite quantification and enzyme activity assays

ATP was quantified using a commercial kit (Beyotime, S0027). NADPH/NADP^+^ and NADH/NAD^+^ ratios were determined with enzymatic cycling kits (Beyotime S0179 and S0175). Citrate and fumarate were measured by colorimetric kits (Sangon D799379; Beyotime, S0517). Citrate synthase and succinate dehydrogenase activities were assayed with commercial kits (Boxbio AKAC007C, AKAC011C). All assays followed the manufacturers’ protocols, and results were normalized to total protein.

### Methanol assimilation and product fermentation

A single colony was inoculated into TB medium and grown overnight to stationary phase. Cells were harvested, washed three times with Mops minimal medium, and inoculated into methanol assimilation medium at OD_500_ = 0.1. Cultures were shaken at 37°C, 250 rpm. Samples were taken every 24 hours for OD_500_, methanol, and xylose. Once exponential phase was reached (OD_500_ ≥ 1), 30 mM xylose was added daily until fermentation end.

### Fed-batch fermentation

A single colony was inoculated into 20 ml of TB (37°C, 250 rpm, 20 hours) for primary seed. This was transferred into 100 ml of seed medium at OD_500_ = 0.1 and incubated for 16 to 20 hours (secondary seed). Fed-batch fermentation was performed in a 5-liter bioreactor (initial volume of 2.5 liters) with secondary seed at OD_500_ = 0.1. Conditions were as follows: 37°C, pH 7.0, agitation 200 to 500 rpm to maintain ∼30% dissolved oxygen (cascade control), aeration 2.0 liter/min. The 96-hour process was monitored by a BODS system (Tmaxtree Biotech) for intelligent feeding. Methanol and xylose were maintained at 3 and 2 g/liter, respectively.

### Formate assimilation

The *acs* gene from *S. typhimurium* and *acdH* from *L. monocytogenes* were integrated into pCDF to generate pCDF-p1-*acs*-*acdH*-p2-*hps*-*phi*. The plasmid was electroporated into the host strain. Single colonies were selected and grown overnight in TB to late exponential/stationary phase. Cells were harvested, washed, and inoculated into formate medium at OD_500_ = 0.1. Formate concentration was measured by high-performance liquid chromatography (HPLC) every 24 hours.

### Glycolate bioconversion

Glycolate bioconversion was performed as described. *hacS* from *C.* bacterium HGW-*Chloroflexi*-9 was cloned into pCDF-Duet (pCDF-*cfh*-*hacS*). Separately, *acr*, *eutE*, and *gldA* were coexpressed from pET-Duet (pET-*acr*-*eutE*-*gldA*). The dual-plasmid strain was induced in TB, harvested, washed, and resuspended in Mops with 10 mM formaldehyde. Glycolate was quantified by HPLC every 24 hours.

### Overexpression of *mreB*(V45R) in *B. methanolicus*

The pTCas9 backbone was amplified from a ThermoCas9-deleted construct. The *mreB*(V45R) fragment (V45R substitution introduced by site-directed mutagenesis) was amplified from *B. methanolicus* genomic DNA. The backbone and fragment were assembled by seamless recombination and transformed into *E. coli* JM109. The verified plasmid pTCas9-pmtlA-*mreB*(V45R) was electroporated into *B. methanolicus*. Positive transformants were screened by colony PCR and cultured in TSB. Expression was induced with 0.5 mM mannitol to activate the pmtlA promoter.

### Statistical methods

All data are presented as mean ± SD from three independent biological replicates (*n* = 3). For comparisons between two groups, a two-tailed unpaired Student’s *t* test was used. For comparisons among three or more groups, one-way analysis of variance (ANOVA) followed by Tukey’s honest significant difference (HSD) post hoc test was applied. Significance levels are indicated as *P* < 0.05, *P < 0.01, **P < 0.001, and ***P < 0.0001 or by lowercase letters (different letters denote significant difference at *P* < 0.05). “n.s.” indicates not significant (*P* > 0.05).
