# Supplementary material for: Engineering bacterial morphology through MreB remodeling enhances one-carbon metabolism for sustainable bioproduction
Source: Sci Adv. 2026 Oct 28;12(44):eaej6280. doi: 10.1126/sciadv.aej6280 (PMC13715707; doi:10.1126/sciadv.aej6280)
Supplement: Supplementary file 1 [file sciadv.aej6280_sm.pdf]

Science Advances
AAAS

Supplementary Materials for

Engineering bacterial morphology through MreB remodeling enhances one-carbon metabolism for sustainable bioproduction

Yun Chen *et al.*

Corresponding author: Zaigao Tan, ztan0918@sjtu.edu.cn

Sci. Adv. **12**, eaej6280 (2026)
DOI: 10.1126/sciadv.aej6280

This PDF file includes:

Figs. S1 to S7
Tables S1 and S2

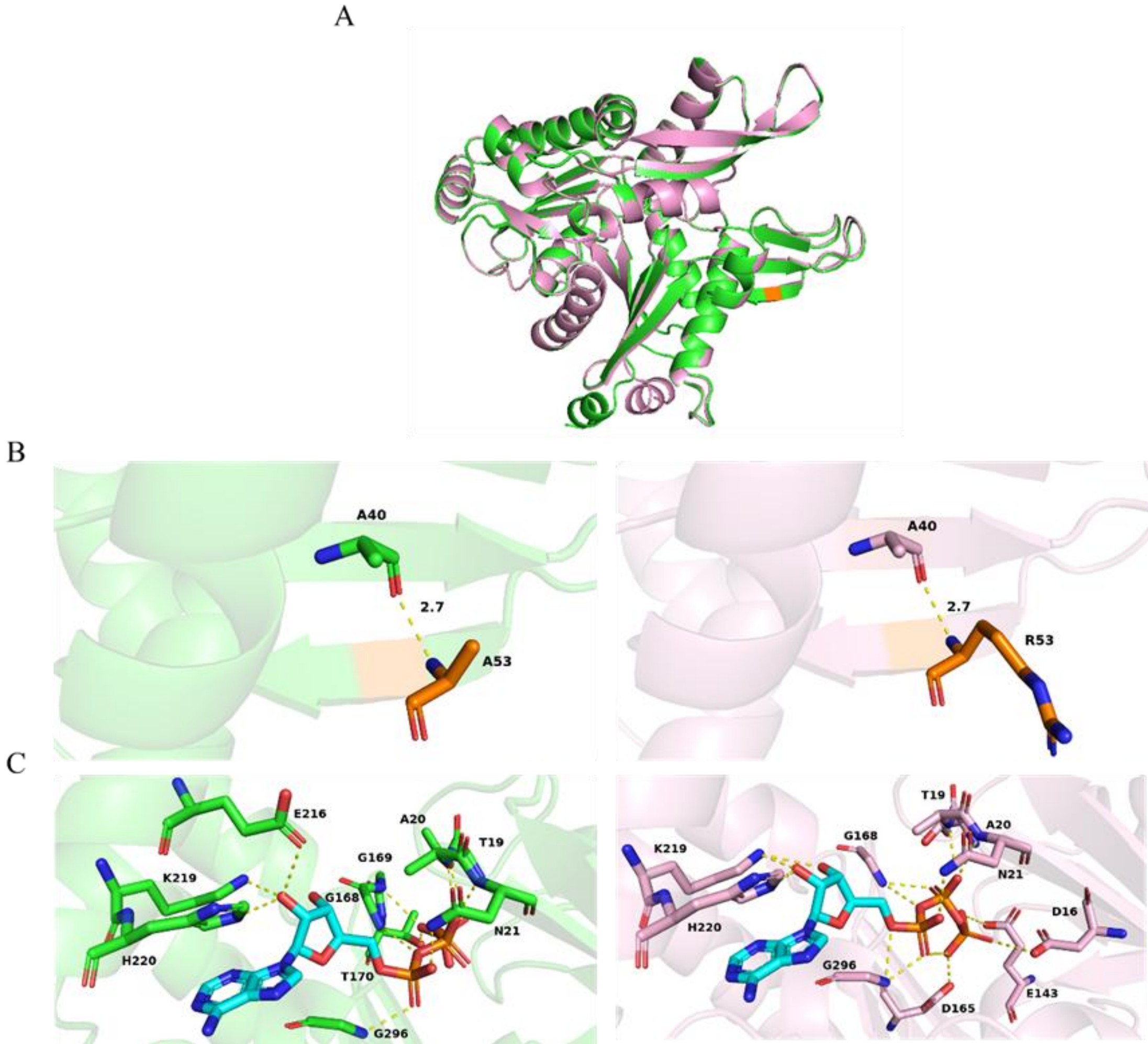

Fig. S1.

Structural analysis of Ec-MreB (A53R) and its ATP-binding affinity.

(A) Superimposition of the protein structures of Ec-MreB (WT) and Ec-MreB (A53R);

(B) Detailed structural comparison of Ec-MreB (WT) and Ec-MreB (A53R);

(C) Detailed molecular docking of ATP with Ec-MreB (WT) and Ec-MreB (A53R), respectively.

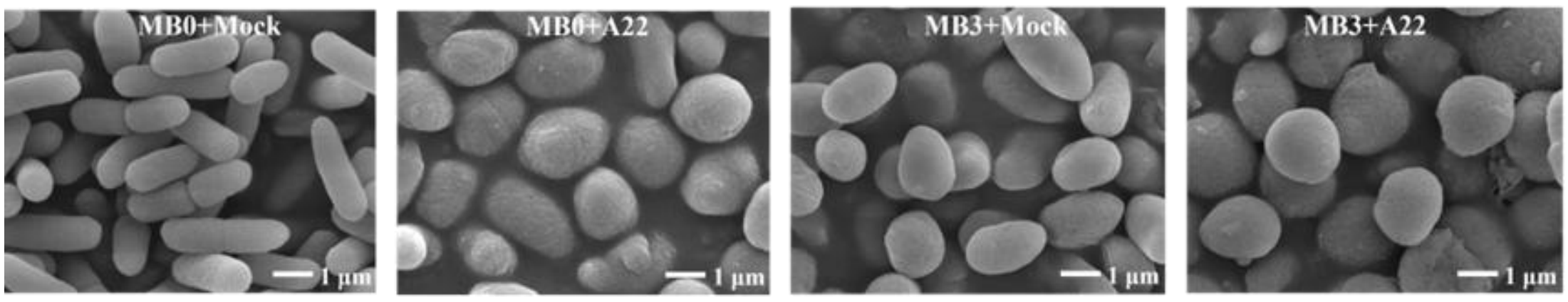

Fig. S2.

SEM figures for MB0+Mock, MB0+A22, MB3+Mock and MB3+A22.

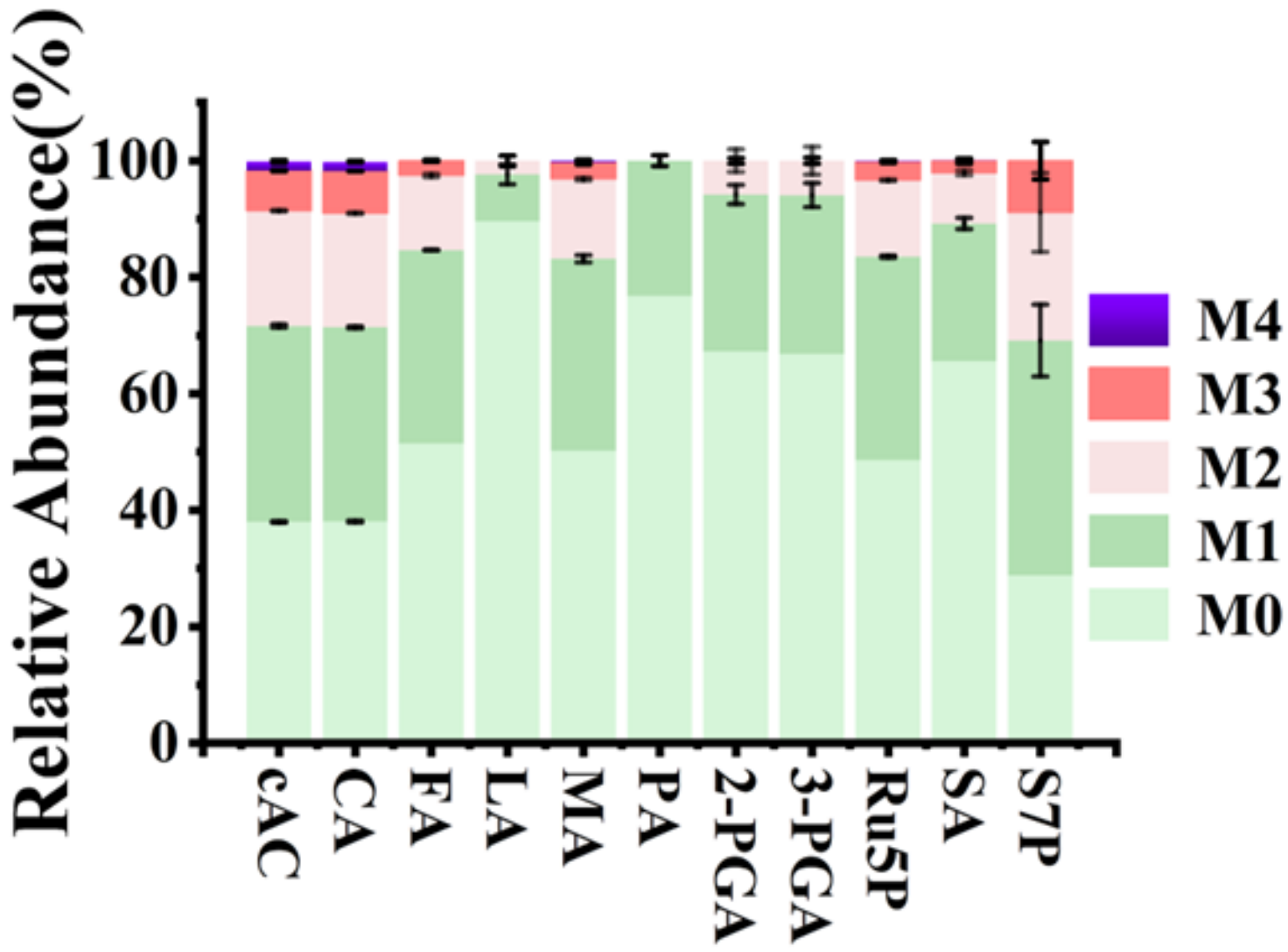

Fig. S3.

Tracing of metabolic flux via ^{13}C-labelled methanol in MB7: key intermediates in the EMP, PPP, and TCA pathways.

Cells were cultured in MOPS medium containing 100 mM ^{13}C-methanol, 50 mM xylose, and 2% casamino acids for 96 h. Bar plots show the percentage of labeled carbon in metabolites of the EMP pathway (2-PGA, pyruvate,3-PGA), PPP (S7P, Ru5P), and TCA cycle (fumarate, malate, succinate, cis-aconitate, citrate,). cAC, cis-aconitic acid; CA, citric acid; FA, fumaric acid; MA, malic acid; 2-PGA, 2-phosphoglyceric acid; 3-PGA, 3-phosphoglyceric acid; PA, pyruvic acid;Ru5P, ribulose-5-phosphate; SA, succinic acid; S7P, Sedoheptulose-7-phosphate.

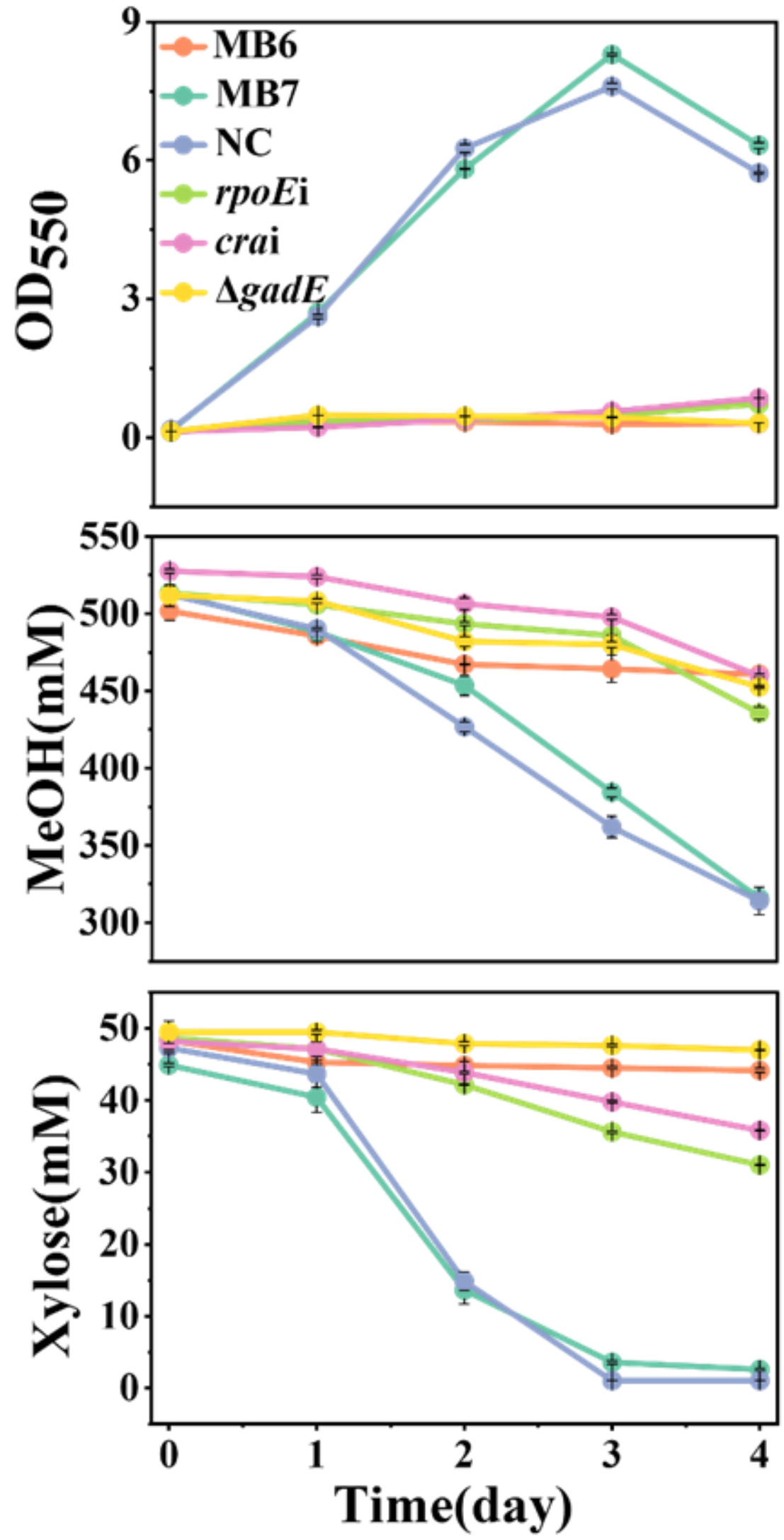

Fig. S4.

Growth (OD_{550}) and substrate consumption (methanol and xylose) of NC, *rpoE*-i, *cra*-i, and deletion of *gadE* (Δ*gadE*) strains.

Cultures were grown in 40 mL MOPS medium containing 450 mM methanol and 50 mM xylose in 250 mL baffled shake flasks. Data are mean ± s.d. (n = 3).

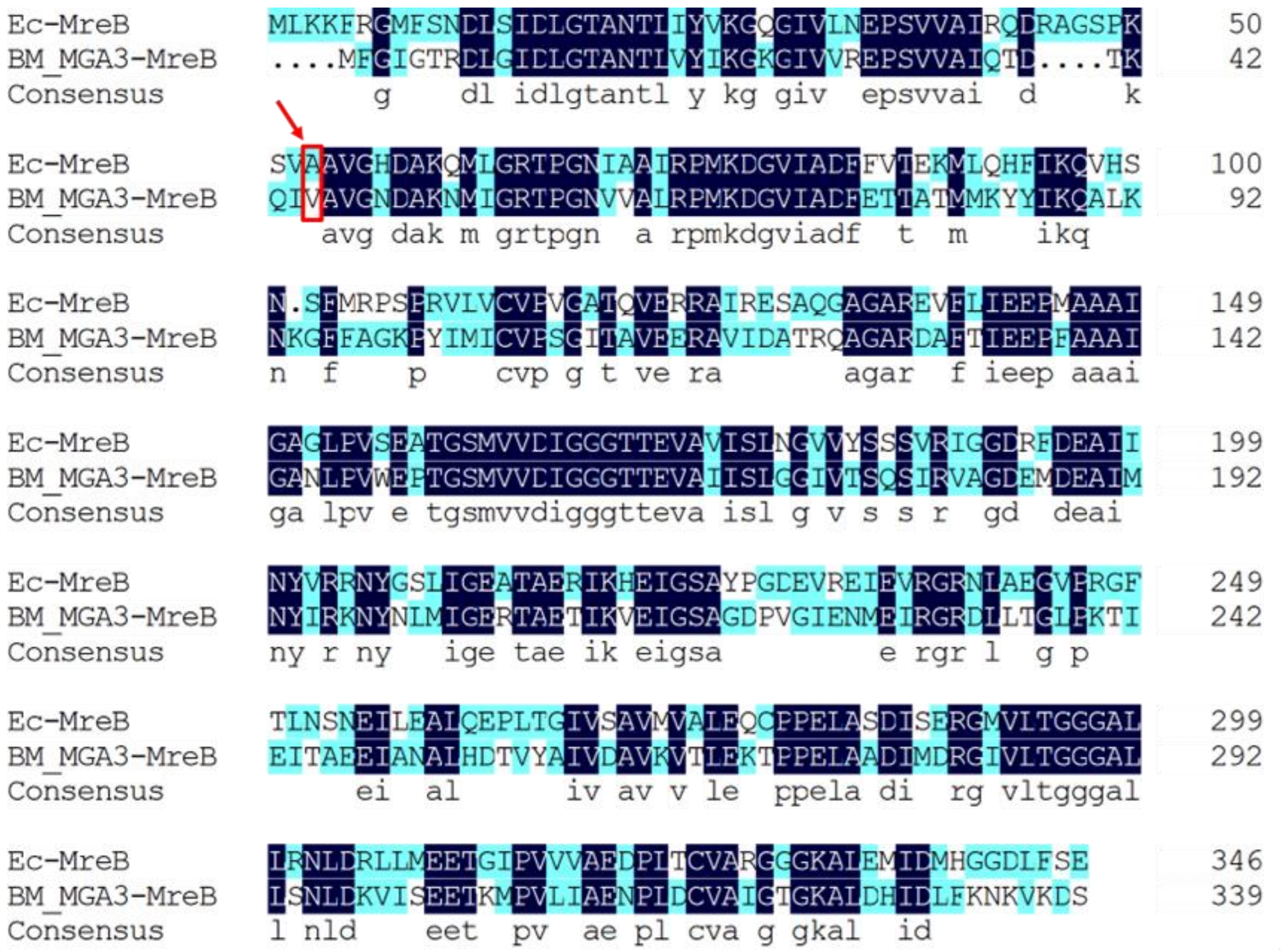

Fig. S5.

Amino acid sequence alignment of Ec-MreB and BM-MreB.

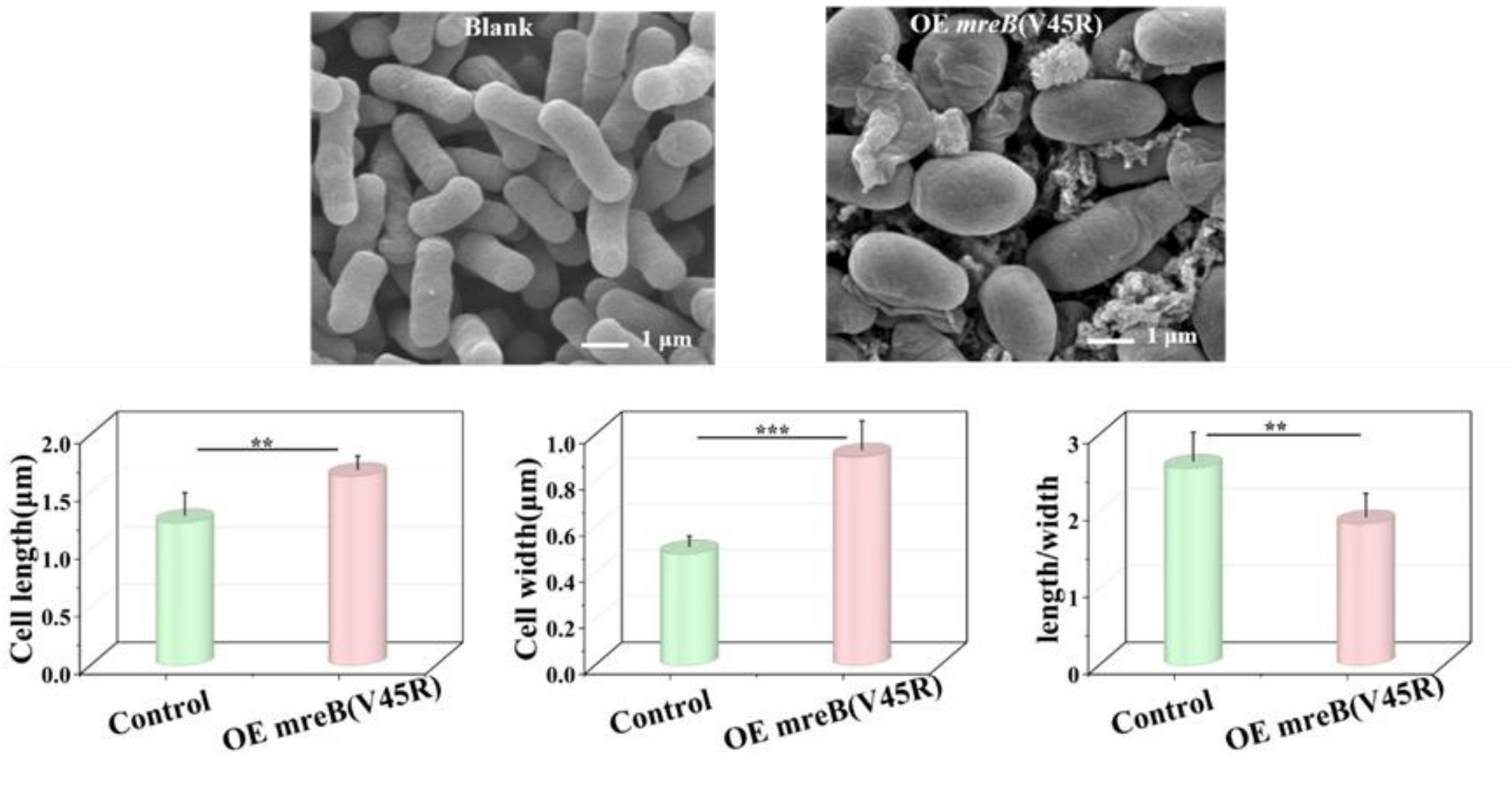

Fig. S6.

SEM figures of *B. methanolicus* MGA3 harboring pTCas-PmtlA (Blank) or *B. methanolicus* MGA3 harboring pTCas-PmtlA-*mreB*$_{(V45R)}$ (OE *mreB*$_{(V45R)}$).

The representative SEM image of the Blank strain is also reused in Fig. S7A as the corresponding control for comparison with the OE *mreB*($_{WT}$) strain.

Cell length and width were measured using ImageJ after calibration with a stage micrometer. “**” means $P \leq 0.01$, “***” means $P \leqslant 0.001$. unpaired two-tailed Student's t-test or appropriate post hoc comparison.

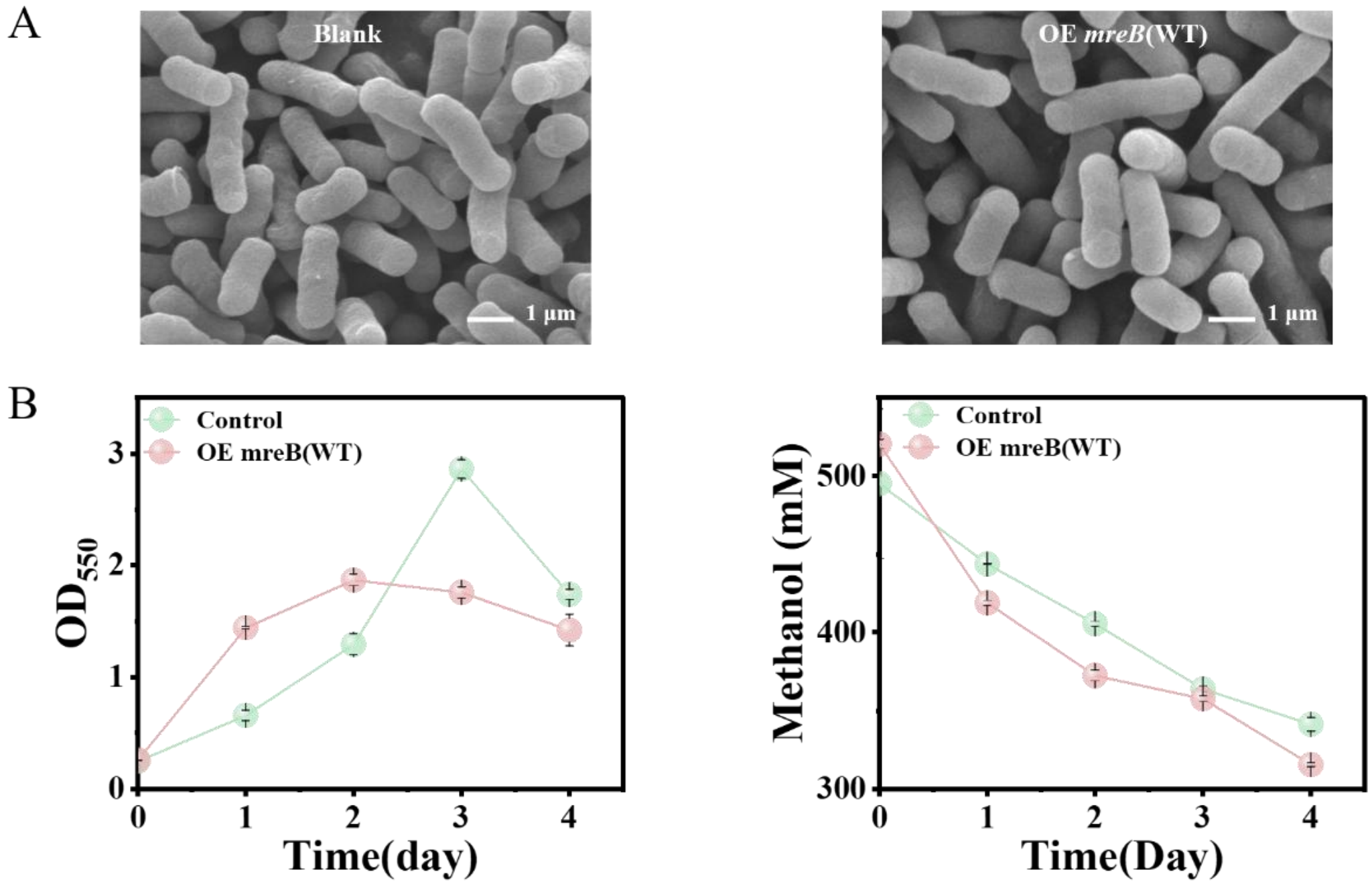

Fig. S7.

SEM figures and methanol utilization of *B. methanolicus* MGA3 harboring pTCas-PmtlA (Blank) or *B. methanolicus* MGA3 harboring pTCas-PmtlA-*mreB*(WT) (OE *mreB*(WT)).

(A) Representative SEM images of *B. methanolicus* MGA3 harboring pTCas-PmtlA (Blank) or *B. methanolicus* MGA3 harboring pTCas-PmtlA-*mreB*(WT) (OE *mreB*(WT)). The Blank SEM image is reproduced from Fig. S6 and is reused here as the corresponding control for comparison with the *mreB*(WT)-overexpressing strain.

(B) Growth curve (OD_{550}) and methanol consumption of wild type MGA3 and OE *mreB*(WT) strains. Cultures were grown in MvcMY minimal medium containing 400 mM methanol and incubated at 50°C in 50 ml conical tubes.

Name	Genotype	Description	Reference
Dh5alpha	F– endA1 glnV44 thi-1 recA1 relA1 gyrA96 deoR nupG purB20 φ80dlacZΔM15 Δ(lacZYA-argF)U169, hsdR17(rK–mK+), λ–	Wild-type used for plasmid construction.	N/A
MG1655	F-, λ-, rph-1	Wild-type used for establishing formaldehyde tolerant strain.	N/A
MG1655(DE3)	K12 Fλ-50 rph-1 (DE3)	Initial strain for methanol assimilation.	N/A
MB0	MG1655Δ*pta*	Control strain for formaldehyde tolerance test.	This study
MB1	MG1655Δ*pta* :: m1-93-*mreB* $_{(WT)}$	Intermediate strain constructed by formaldehyde tolerant engineering strain.	This study
MB2	MG1655Δ*pta* :: m1-93-*mreB* $_{(A53R)}$	Engineered strain for formaldehyde tolerance test.	This study
MB3	MB2 Δ *mreB* $_{(WT)}$	Engineered strain for formaldehyde tolerance test.	This study
MB4	MG1655(DE3)Δ*pta*Δ*frmA*Δ*rpiAB* Δ*cyaA*	Control chassis strain for methanol assimilation test.	This study
MB5	MG1655(DE3)Δ*pta*::m1-93-*mreB* $_{(A53R)}$Δ*mreB* $_{(WT)}$Δ*frmA*Δ*rpiAB*Δ*cyaA*	Engineered chassis strain for methanol assimilation test.	This study
MB6	MB4 harboring pCDF-RuMP vector	Control strain for methanol assimilation test.	This study
MB7	MB5 harboring pCDF-RuMP vector	Engineered strain for methanol assimilation test.	This study
MB16	MB5 harboring pCDF-*StACS*-*LmACDH2*-P2-*hps*-*phi* vector	Control strain for formate assimilation test.	This study
MB17	MB6 harboring pCDF-*StACS*-*LmACDH2*-P2-*hps*-*phi* vector	Engineered strain for formate assimilation test.	This study
MB18	MB5 harboring pET-*acr*-*eutE*-*gldA* and pCDF-*hacl* vector	Control chassis strain for glycolate bioconversion from formaldehyde through FORCE pathway.	This study
MB19	MB6 harboring pET-*acr*-*eutE*-*gldA* and pCDF-*hacl* vector	Engineered chassis strain for glycolate bioconversion from formaldehyde through FORCE pathway.	This study
OE *mreB* $_{(V45R)}$	Bacillus methanolicus harboring pTCas-PmtlA-*mreB* $_{(V45R)}$ vector	Engineered chassis strain for *B. methanolicus* shape-altering and methanol consumption test.	This study

Table S1.

Strain list.

Name	Description	Reference
pEcCas	Plasmid for CRISPR with λRed recombinase system, Kan^R	Lab collection
pEcgRNA-*pta*	pTargetF plasmid for deletion of *pta* and integrate m1-93-*ftsZ*, $Spec^R$	This study
pEcgRNA-*frmA*	pTargetF plasmid for deletion of *frmA*, $Spec^R$	This study
pEcgRNA-*rpiA*	pTargetF plasmid for deletion of *rpiA*, $Spec^R$	This study
pEcgRNA-*rpiB*	pTargetF plasmid for deletion of *rpiB*, $Spec^R$	This study
pKD46	Plasmid for providing λRed homologous recombination systems, temperature-sensitive replicons (30 °C), $Carb^R$	Lab collection
pCDF-RuMP	pCDF-Duet with *Cupriavidus necator mdh* gene in the P1 cloning site and *Bacillus methanolicus hps* and *phi* genes in the P2 cloning site under T7 promoter, $Spec^R$	Lab collection
pdCas9-bacteria	aTc-inducible expression of a catalytically inactive bacterial Cas9 (*S. pyogenes*) for bacterial gene knockdown	Novopro
pEcgRNA-*rpoE*	pTargetF plasmid for *rpoE* knockdown	This study
pEcgRNA-*cra*	pTargetF plasmid for *cra* knockdown	This study
pEcgRNA-*gadE*	pTargetF plasmid for *gadE* knockout	This study
pCDF-*Stacs*-*Lmacdh2*-P2-*hsp*-*phi*	pCDF-Duet with *acs* from *Salmonella typhimurium*, the *acdh2* gene from *Listeria monocytogenes* in P1 cloning site and *Bacillus methanolicus hps* and *phi* genes in the P2 cloning site under T7 promoter, $Spec^R$	This study
pET-*acr-eutE-gldA*	pET-Duet with *Listeria monocytogenes acr*, *Salmonella typhimurium eutE* and *Escherichia coli aldA* genes under CT5 promoter, $Carb^R$	Lab collection
pCDF-*hacl*	pCDF-DueT with *Chloroflexi* bacterium HGW-*Chloroflexi*-9 *hacS* in the P1 cloning site under T7 promoter, $Spec^R$	Lab collection
pTCas9	Template for the editing plasmid of *B. methanolicus* MGA3	43
pTCas9-PmtlA-*mreB*$_{(V45R)}$	pTCas9 plasmid with *cas9* deleted and *B. methanolicus mreB*$_{(V45R)}$ integrated	This study

Table S2.

Plasmids used or developed in this study
